# Real-Time Biosynthetic Reaction Monitoring Informs the Mechanism of Action of Antibiotics

**DOI:** 10.1021/jacs.4c00081

**Published:** 2024-03-01

**Authors:** Abraham O. Oluwole, Víctor M. Hernández-Rocamora, Yihui Cao, Xuechen Li, Waldemar Vollmer, Carol V. Robinson, Jani R. Bolla

**Affiliations:** † Department of Chemistry, University of Oxford, South Parks Road, Oxford OX1 3QZ, U.K.; ‡ The Kavli Institute for Nanoscience Discovery, University of Oxford, South Parks Road, Oxford OX1 3QU, U.K.; § Centre for Bacterial Cell Biology, Biosciences Institute, Newcastle University, Richardson Road, Newcastle upon Tyne NE2 4AX, U.K.; ∥ Department of Chemistry, State Key Laboratory of Synthetic Chemistry, The University of Hong Kong, Pokfulam Road, Hong Kong SAR 999077, China; ⊥ Institute for Molecular Bioscience, University of Queensland, Carmody Road, Brisbane, Queensland 4072, Australia; # Department of Biology, University of Oxford, South Parks Road, Oxford OX1 3RB, U.K.

## Abstract

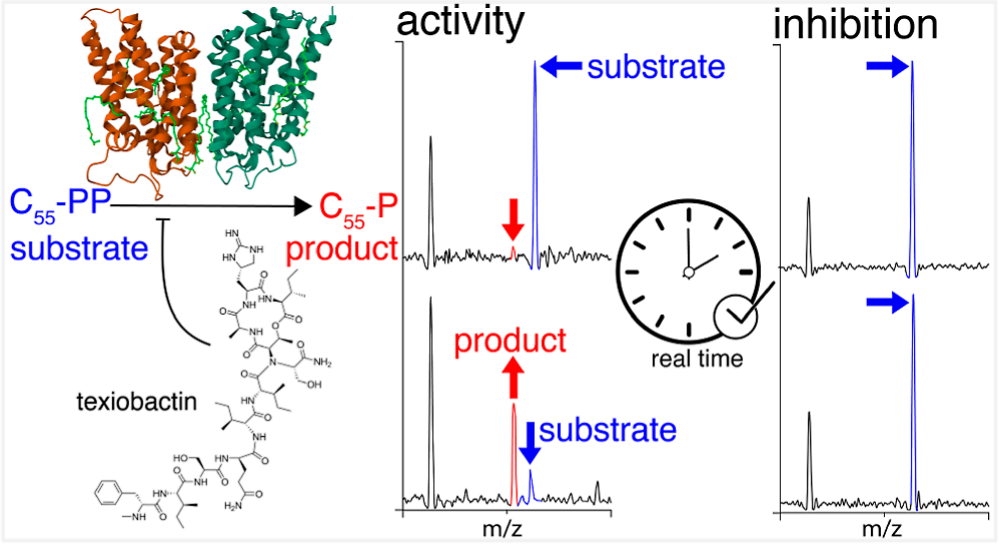

The rapid spread of drug-resistant pathogens and the declining discovery of new antibiotics have created a global health crisis and heightened interest in the search for novel antibiotics. Beyond their discovery, elucidating mechanisms of action has necessitated new approaches, especially for antibiotics that interact with lipidic substrates and membrane proteins. Here, we develop a methodology for real-time reaction monitoring of the activities of two bacterial membrane phosphatases, UppP and PgpB. We then show how we can inhibit their activities using existing and newly discovered antibiotics such as bacitracin and teixobactin. Additionally, we found that the UppP dimer is stabilized by phosphatidylethanolamine, which, unexpectedly, enhanced the speed of substrate processing. Overall, our results demonstrate the potential of native mass spectrometry for real-time biosynthetic reaction monitoring of membrane enzymes, as well as their in situ inhibition and cofactor binding, to inform the mode of action of emerging antibiotics.

## Introduction

Over the past decade, there has been a significant increase in the search for novel antibiotics, using innovative methodologies ranging from the ability to cultivate previously uncultured organisms to advancements in artificial intelligence.^1−5^ These advancements are considerably enhancing the pace of antibiotic discovery,^6−10^ including those that are active against Gram-positive bacteria without detectable resistance, for example, teixobactin,^7^ and those that are active against Gram-negative pathogenic bacteria, for example, darobactin.^9^ A major hurdle in translating these newly identified molecules into clinical antibiotics, however, is the task of elucidating their mechanisms of action (MoA). Such an understanding can also foster precision therapeutics, resistance abatement strategies, and the development of new analogues.

Methods for characterizing the MoA of antibiotics, including affinity chromatography and photoaffinity labeling, often require ligand immobilization or chemical modification of the drug and are unable to detect weak interactions.^11^ Alternative methods such as drug affinity responsive target stability^12^ and thermal shift assays,^13^ which take advantage of the changes in protein thermostability in response to drug binding, can be used to identify targets that directly interact with unmodified drugs. Omics approaches (genomics, transcriptomics, proteomics, and metabolomics)^14−17^ can probe phenotypic changes in the cell in response to antibiotic stimuli. In addition to these methodologies, native mass spectrometry (native MS) can capture noncovalent interactions between proteins and substrates, lipids and drugs, presenting a new platform for probing the MoA of antibiotics.^18,19^ By leveraging signal intensities as a measure of the relative abundance of species in solution, native MS reports on protein–ligand binding affinity,^20−23^ and enzyme catalysis.^24,25^

We aimed to develop a combination of real-time enzyme activity monitoring and in situ inhibition to yield a mechanistic insight into the MoA of antibiotics. To develop our approach, we selected two *Escherichia coli* membrane phosphatases UppP and PgpB as model membrane enzymes, having different substrate specificities.^26,27^ These membrane enzymes are involved in carrier lipid metabolisms which is important for the biosynthesis of peptidoglycan and other cell envelope polymers.^28^

**Figure 1:**
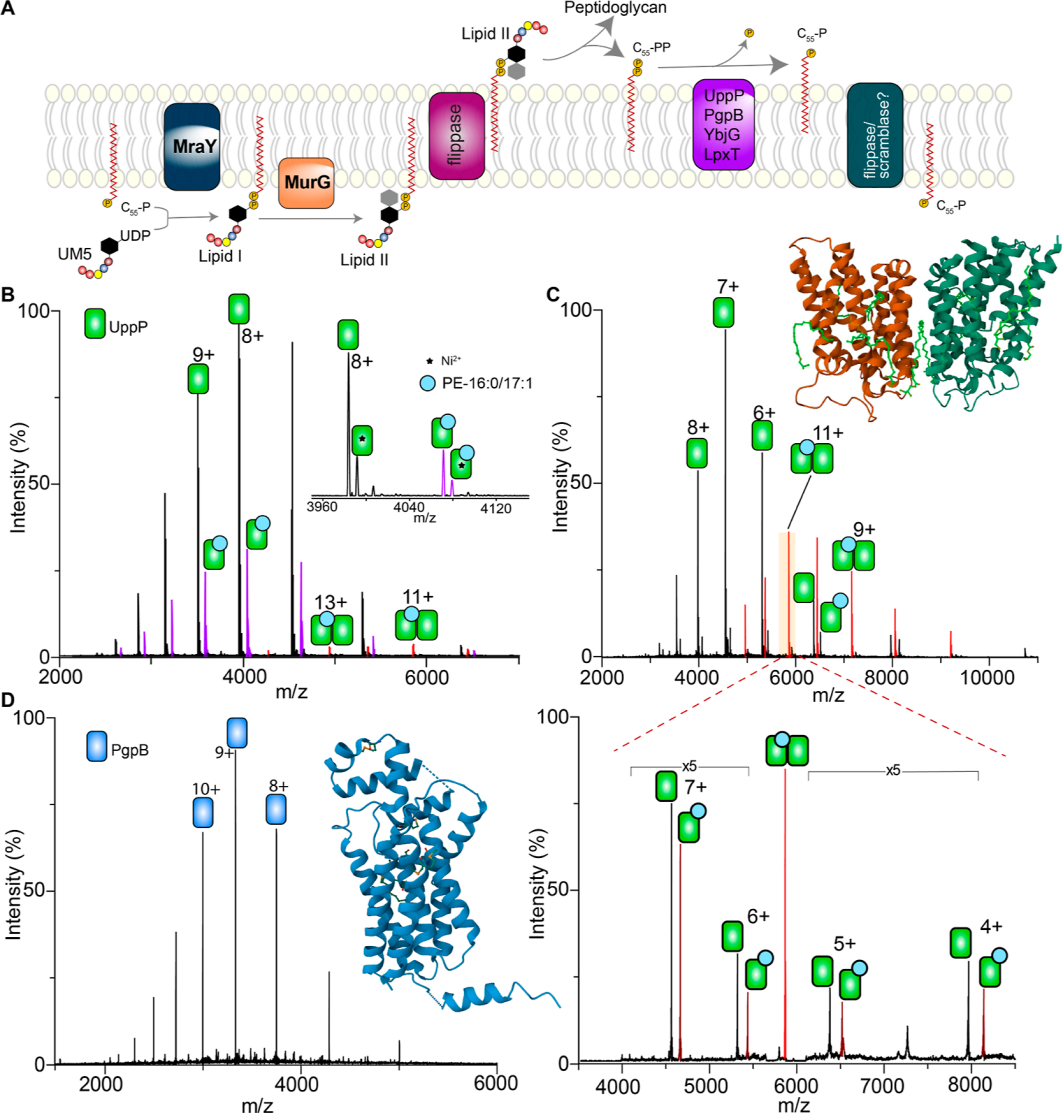
Distinct lipid interactions of UppP and PgpB. (A) Schematic illustration of the peptidoglycan synthesis pathway in *E. coli*, highlighting the central role of the undecaprenyl pyrophosphate (C_55_-PP) phosphatase enzymes (purple). The cytosolic precursor, uridine diphosphate *N*-acetylmuramyl-pentapeptide (UM5), reacts with C_55_-P to form lipid I through the action of MraY and is then glycosylated by MurG to form lipid II. Following lipid II flipping into the periplasm, where the glycopeptide moiety is incorporated into the nascent peptidoglycan, the carrier lipid is released in the form of a diphosphate (C_55_-PP). C_55_-PP is then dephosphorylated by UppP or the PAP2-type phosphatases (PgpB, LpxT, and YbjG) and then flipped such that the phosphate head faces the cytosol to re-enter the pathway. (B) Mass spectrum of UppP released from LDAO micelles using collisional activation of 100 V. Peaks corresponding to apo monomer (black), lipid-bound monomer (purple), and lipid-bound dimer (red) are observed. (Insert) a zoomed view of 8+ charge state. Adducts correspond to copurified Ni^2+^. (C) Equivalent spectrum recorded with higher collisional activation (180 V) enhanced the intensities of peaks assigned to the lipid-bound UppP dimer. Tandem MS on a lipid-bound dimer charge state (11+) yielded a mixed population of apo- and lipidated protomers. Peaks are assigned to UppP monomers in the apo form and those bound to phospholipids (highlighted in purple in the tandem MS). (Insert) X-ray structure of UppP (PDB code 6CB2). (D) Mass spectrum of *E. coli* PgpB released from LDAO micelles using similar activation conditions (100–200 V) exhibited peaks corresponding to monomeric protein only with little to no phospholipid adducts. (Insert) X-ray structure of PgpB (PDB code 4PX7).

Synthesis of peptidoglycan, a highly conserved component of the bacterial cell wall and the target of many successful antibiotics,^29^ begins in the cytosol with the synthesis of disaccharide pentapeptide precursors^30^ which are then attached to C_55_-P by MraY to form lipid I.^31^ Lipid I is subsequently glycosylated into lipid II by MurG,^32^ and then transported across the membrane by flippases such as MurJ and Amj.^33−35^ The carrier lipid is finally released as undecaprenyl diphosphate (C_55_-PP) after the glycopeptide moieties are incorporated into the nascent peptidoglycan polymer by the penicillin-binding proteins and other machineries (Figure 1A).^36−38^ C_55_-PP is also synthesized de novo but must first be dephosphorylated before it can be used for translocating cell wall precursors.^39^ Carrier lipid molecules are dephosphorylated by phosphatases of the UppP or PAP2 families,^40,41^ and are transported back across the plasma membrane by DedA and DUF368 family proteins^28,42,43^ before re-entering the lipid II cycle.^44^ Most of the antibiotics that interfere with this peptidoglycan biosynthesis pathway appear to specifically act by sequestering the key membrane-bound intermediates, namely, C_55_-P, C_55_-PP, lipid-I, and lipid-II, rather than by targeting the enzymes themselves. This MoA is exemplified by amphomycin, bacitracin, nisin, ramoplanin, and vancomycin.^45^ Since the targeted precursors are nonproteinaceous in nature, they are not easily mutated or modified. Consequently, they continue to hold promise for antibiotic discovery and development.

In *E. coli,* four enzymes dephosphorylate C_55_-PP in the periplasm, namely UppP and the three PAP2 proteins—PgpB, YbjG, and LpxT. UppP (also known as BacA) is the main C_55_-PP phosphatase in *E. coli*, providing up to 75% of this activity.^46^ The PAP2 enzyme PgpB is the main contributor to the last step of phosphatidyl–glycerol synthesis by dephosphorylating diacylglycerol phosphate.^27^ The absence of PgpB can also impair glycosyltransferase and transpeptidase activities of PBP1B, and thus, PG synthesis.^47^ Using C_55_-PP as a phosphate donor, LpxT can phosphorylate the lipopolysaccharide precursor lipid A^48^ but LpxT alone cannot supply the essential C_55_-PP dephosphorylation function required for cell growth.^46^

Here we develop a native MS approach to demonstrate that the enzymatic activities of UppP and PgpB can be monitored in real time. We then used this method to explore substrate specificities and to inhibit their activities in situ using existing and newly discovered antibiotics. We captured the impact of canonical active site residues on the speed of UppP catalysis and found that the kinetics of substrate processing by UppP is enhanced by phosphatidylethanolamine (PE) lipids. By analyzing UppP in a range of solution and MS conditions we uncover a previously unknown phospholipid requirement for UppP dimerization. PgpB homologs exhibit a lower affinity for endogenous phospholipids than UppP and do not form lipid-mediated oligomers. We provide direct evidence that bacitracin and teixobactin do not only sequester free substrates in solution but can also outcompete UppP and PgpB for substrates. Overall, our results demonstrate the potential of native MS, as a powerful complement to existing biochemical tools for investigating the MoA of antibiotics, specifically for real-time biosynthetic reaction monitoring and in situ inhibition of membrane enzymes.

## Results and Discussion

### Distinct Lipid-Binding Properties of UppP and PgpB

We first expressed and purified UppP and PgpB from membrane fractions of *E. coli* and performed native MS by releasing protein ions from a buffer containing 200 mM ammonium acetate (pH 8.0) and 0.05% LDAO. The native mass spectrum of UppP reveals that the protein exists in an apo form (31,861.73 ± 0.23 Da, expected mass 31,861.97 Da) and in complex with ligand species (32,562.5 ± 1.23 Da), which are potentially phospholipids (Figure 1B). To identify the bound ligands, we performed tandem MS by selecting and activating a peak assigned to a ligand-bound monomer. This yielded a peak corresponding to the apo monomer, reflecting the loss of a ∼702-Da species (Figure S1A). After performing lipid extraction from this protein and recording a mass spectrum, we observed a range of phospholipids, including the ∼702-Da species (Figure S1B). Performing MS/MS on the 702-Da species yielded fragments consistent with PE, predominantly PE(16:0/17:1) (Figure S1B), indicating that PE might be important for the structural integrity of UppP.

To test this hypothesis, we recorded UppP spectra using 200 V in the high-energy collision-induced dissociation cell, allowing higher-order species to be transmitted. Our data show the presence of additional peaks corresponding to UppP dimers in complex with PE (Figure 1C). To understand how detergent may affect UppP dimerization and lipid binding, we screened several additional detergents and recorded spectra at a range of protein concentrations (Figure S2). In all cases, we observe UppP dimers in complex with endogenous PE, indicating a high-affinity binding interaction. It is intriguing to note that in all cases little-to-no lipid-free UppP dimer was detected. This suggests a stabilizing role of PE, in accordance with an earlier report that UppP is able to form dimers.^40^ Taken together, our data uncover a previously unknown phospholipid requirement for UppP dimerization.

The native mass spectrum of *E. coli* PgpB (PgpB^Ec^), by contrast, exhibited peaks assigned primarily to the apo monomer (30,025.57 ± 0.53 Da, expected mass 30,026.27 Da) with little or no phospholipid adducts (Figure 1D). We probed this observation further by equilibrating PgpB^Ec^ with fourfold molar excess of exogenous POPE and recorded native MS. The spectrum exhibited peaks assigned apo- and POPE-bound PgpB^Ec^ monomer (Figure S3), but no dimerization was observed. The use of less stringent purification conditions (see the Methods section) yielded monomeric PgpB^Ec^ in apo and cardiolipin-bound forms rather than PE (Figure S3). Additionally, we expressed *Bacillus subtilis* PgpB (PgpB^Bs^) in *E. coli* and analyzed the purified protein by native MS. The spectrum exhibited peaks consistent with the expected mass of monomeric PgpB^Bs^ (25,890.75 ± 0.57 Da, expected mass 25,891.65 Da) but without copurified phospholipids (Figure S3). Taken together, these data suggest that both homologs of PgpB have a considerably lower affinity for membrane phospholipids than UppP and do not form lipid-mediated oligomers.

### Monitoring the Enzymatic Activities of UppP and PgpB in Real Time

Having optimized the native MS conditions for UppP and PgpB, we next assessed the activities of the purified proteins. For this, we incubated 3.5 μM of delipidated UppP with 50 μM farnesyl diphosphate, C_15_-PP. The latter is more commonly used as model substrate in the in vitro assays for phosphatase function^27,49^ and yielded better spectral quality when used at higher substrate/protein ratios in our MS assays. We then recorded spectra as a function of incubation time using the transition from the micellar solution to the gas phase to quench the reaction. The spectrum recorded after ∼30 s of incubating C_15_-PP with UppP exhibits peaks corresponding to ligand-free UppP and multiple enzyme–substrate complexes (UppP)(C_15_-PP)_*n*_ (Figure S4). After ∼1 min, the protein-bound product C_15_-P was detected. Concomitantly the peak intensity of the (UppP)(C_15_-PP)_*n*_ complexes had decreased. Together these data capture C_15_-PP processing into the C_15_-P product; the latter having a lower binding affinity, therefore exiting the active site to free up the enzyme for the next round of catalysis.

**Figure 2:**
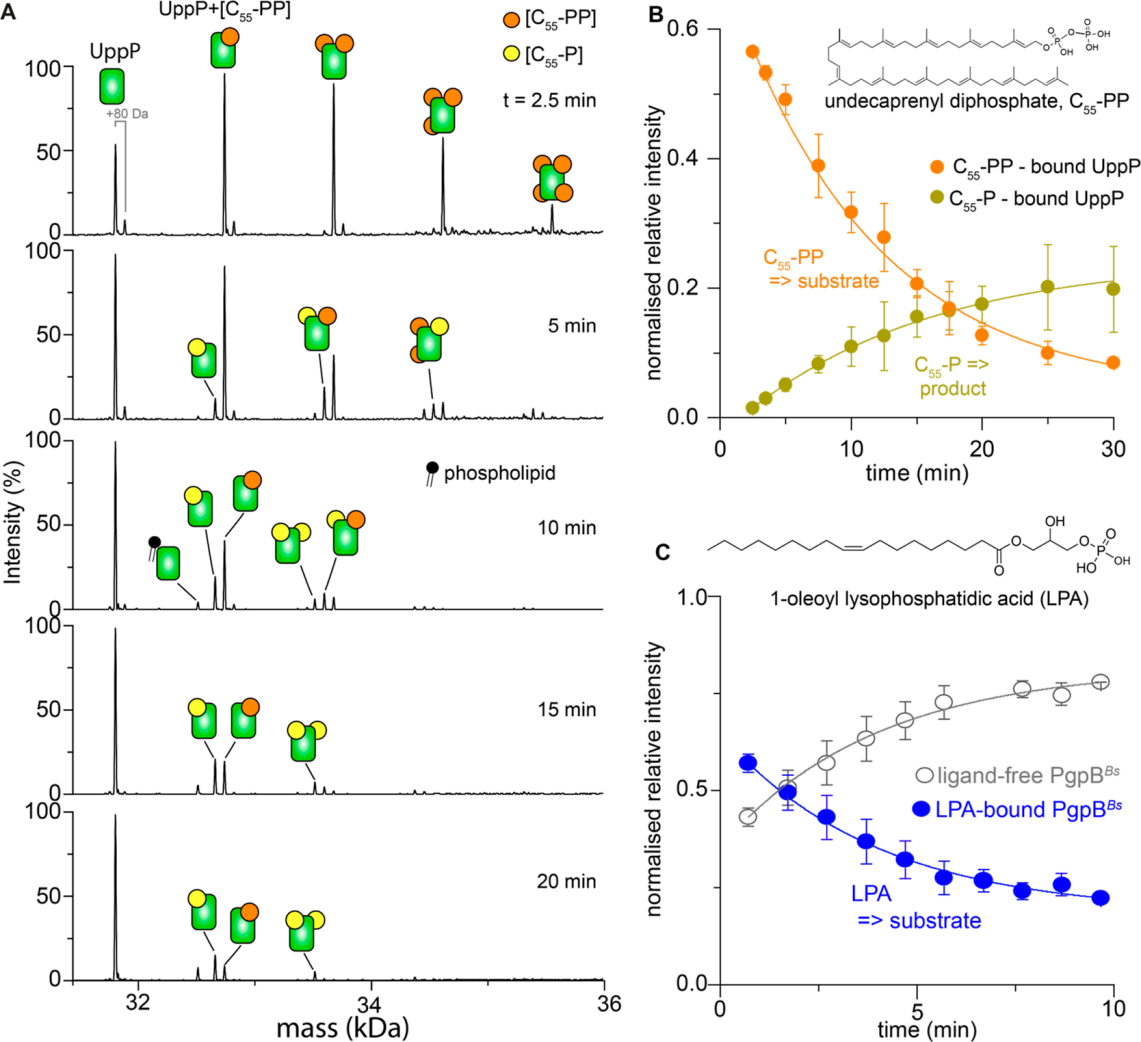
Real-time enzymatic activity of UppP and PgpB. (A) Native mass spectra (deconvoluted) recorded from a solution of 5 μM *E. coli* UppP and 20 μM C_55_-PP monitored as a function of time. The reaction was performed in a buffer containing 200 mM ammonium acetate (pH 8.0), 0.05% LDAO, and 25 μM EDTA. Peaks corresponding to UppP in the apo form (31,862 Da) and those bound to the substrate C_55_-PP (+927 Da) and product C_55_-P (+847 Da) are labeled. Low-intensity peaks (+80 Da) can be assigned to phosphate adducts or the phosphoenzyme intermediate. (B) The relative intensities of UppP-bound C_55_-PP and C_55_-P as a function of time. For this analysis, only the binding of one substrate or one product molecule is considered. Data points are represented by circles; error bars are standard deviations of three replicate measurements. Lines are exponential fit, yielding apparent rate constants 0.090 ± 0.008 min^–1^ and 0.069 ± 0.004 min^–1^ for C_55_-PP and C_55_-P, respectively. (C) Time-course of relative intensities of ligand-free and LPA-bound (+425 Da) PgpB (*B. subtilis*) from the spectra recorded for 5 μM *B. subtilis* PgpB equilibrated with 20 μM 1-oleoyl lysophosphatidic acid (LPA).

Using the same reaction monitoring approach, we explored the activities of UppP toward its longer-chain physiological substrate C_55_-PP. We found that in this case, the reaction is completed at a faster time scale than for C_15_-PP such that the initial enzyme–substrate complex is not captured in the earliest time (30 s); only the C_55_-P product was observed in complex with the protein (Figure S4). In the presence of 200 μM EDTA, the substrate remains bound to the enzyme throughout the reaction time window, but no product was observed (Figure S4). To capture the processing of C_55_-PP by wild-type UppP in real-time, we performed the assay in the presence of 20 μM EDTA to decrease the concentration of divalent cations in the reaction mixture. The resulting spectra exhibited peaks assigned to UppP-bound C_55_-PP at ∼2.5 min (Figure 2A). Peaks corresponding to the dephosphorylated product C_55_-P are observed in the spectra acquired after 5 min of the reaction. Accordingly, the relative intensity of peaks assigned to UppP-bound C_55_-PP decreased while the corresponding peaks for the product C_55_-P increased throughout the time course of the reaction (Figure 2B). Moreover, no further phosphorolysis of C_55_-P into undecaprenol was detected, which is consistent with the specificity of the UppP reaction.

To further confirm the substrate specificities of UppP, we recorded spectra for UppP incubated with 1,2-dioleoylglycerol diphosphate (DGPP), a model substrate of PgpB. The resultant spectra are dominated by peaks assigned to the bound substrate. Peaks corresponding to 1,2-dioleoylglycerol monophosphate (DGP) can be detected, but residual substrates remain, even after prolonged incubation (Figure S4). This observation indicates that UppP preferentially and efficiently processes C_55_-PP over DGPP.

Next, we applied our native MS strategy to PgpB^Ec^ whose activity can be probed using a range of substrates, including phosphatidyl glycerol phosphate (PGP), lysophosphatidic acid (lyso-PA), DGPP, and C_55_-PP.^26,27,50^ We studied the specificity of PgpB^Ec^ toward a phospholipid substrate mimic DGPP, versus the peptidoglycan lipid substrate C_55_-PP. Spectra of a solution containing 10 μM C_55_-PP and 3.5 μM PgpB^Ec^ were recorded following different incubation times. At the onset of the reaction (∼30 s), we observe intense binding of C_55_-PP molecules to the enzyme (Figure S5). However, the peak intensity of the protein-bound product (C_55_-P) is very low (Figure S5). By contrast, the spectrum of 10 μM DGPP incubated with PgpB^Ec^ exhibited intense peaks corresponding to the enzyme–substrate and enzyme–product complexes with stoichiometries ranging from 1:1 to 1:4 (Figure S5). The spectrum recorded after 2 min is dominated by the enzyme–product complex (Figure S5). The presence of a ternary complex formed by PgpB^Ec^ with the substrate DGPP and the monophosphorylated product DGP suggests that the enzyme can coordinate simultaneously both processed and unprocessed lipid molecules. Overall, these data indicate that PgpB preferentially processes a phospholipid precursor DGPP over the carrier lipid C_55_-PP. This ability to discriminate the preferred substrates of different membrane enzymes, which catalyze similar reactions, can advance drug discovery processes by informing the development of chemical moieties tailored to outcompete specific enzyme–substrate interactions.

We next examined the activity of PgpB of *B. subtilis* (PgpB^Bs^) toward DGPP and C_55_-PP for comparison with the *E. coli* homolog. The spectra acquired within 1 min of equilibration exhibited peaks assigned to enzyme–product complexes with no residual substrate (Figure S6). This result indicates a higher efficiency of PgpB^Bs^ over its *E. coli* homolog. We further tested the activity of PgpB^Bs^ toward 1-oleoyl lysophosphatidic acid (LPA). To this end, we recorded spectra for a mixture of 3.5 μM PgpB^Bs^ and 50 μM LPA. This relatively high lipid/substrate molar ratio is required to observe a strong binding intensity to the protein. The resulting spectra exhibited a progressive loss of protein-bound LPA as a function of time (Figure S6). Accordingly, the relative peak intensities of the LPA-bound protein decreased, while those for the ligand-free enzyme increased (Figure 2C). This observation can be attributed to the enzymatic conversion of LPA into 1-oleoylglycerol, with the latter lacking detectible binding affinity to the enzyme. Thus, the *E. coli* and *B. subtilis* PgpB are similar to the *Saccharomyces cerevisiae* diacylglycerol pyrophosphate phosphatase 1 (DPP1) in their ability to dephosphorylate DGPP and LPA.^51,52^

### Differential Effect of Divalent Cations on UppP and PgpB Function

**Figure 3:**
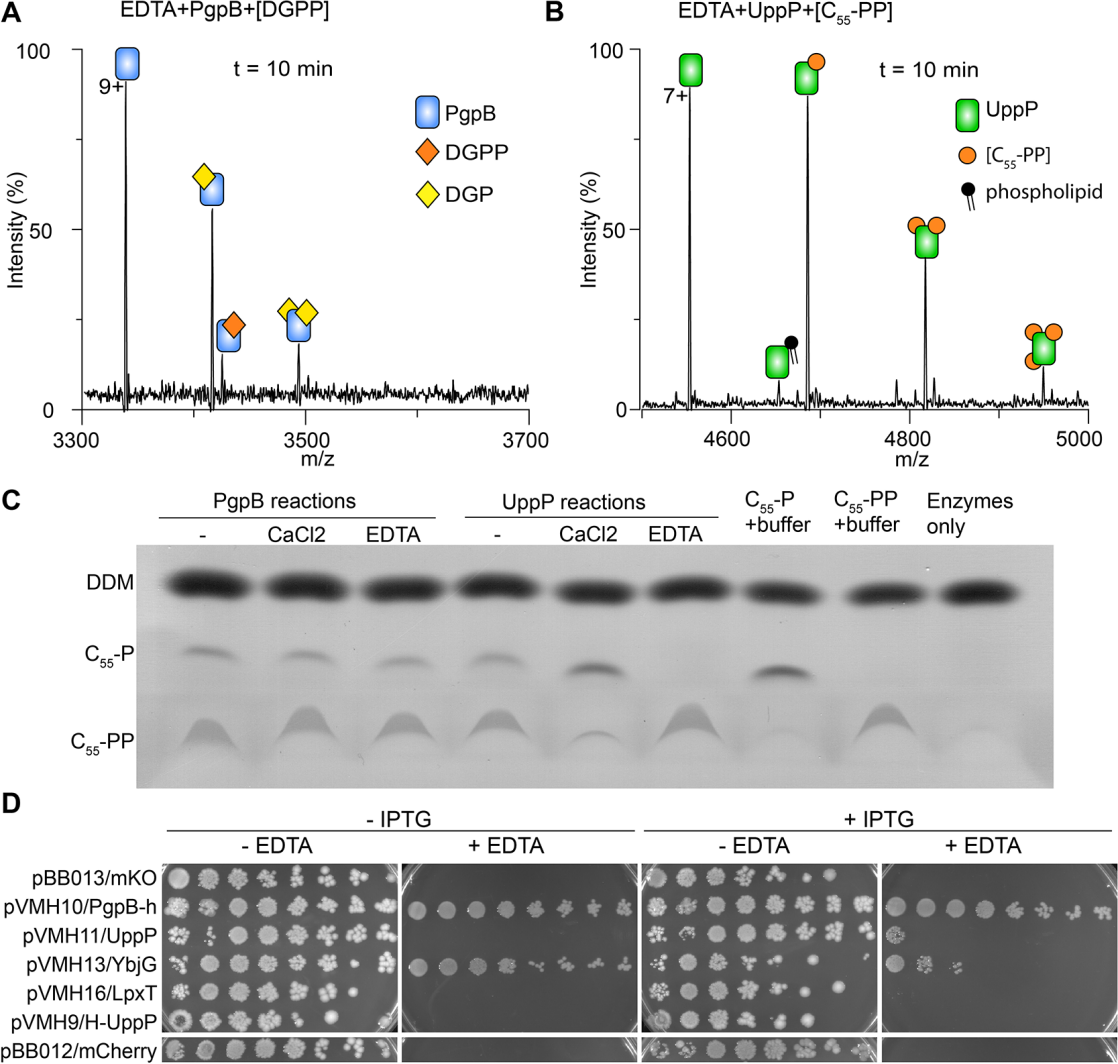
Substrate selectivity of PgpB and divalent cation dependence. (A) Spectrum for 3.5 μM PgpB and 10 μM DGPP in the presence of 100 μM EDTA. (B) Spectrum for 3.5 μM UppP and 10 μM C_55_-PP in the presence of 100 μM EDTA. For UppP, catalysis failed in the presence of EDTA due to the absence of divalent cations. (C) TLC analysis of reaction products of C_55_-PP phosphatase assays with PgpB and UppP, using iodine as a stain. UppP is activated by CaCl_2_ and inhibited by EDTA whereas PgpB activity is not affected by either CaCl_2_ or EDTA. One micromole of each enzyme was incubated with 35 μM C_55_-PP in the presence or absence of 10 mM CaCl_2_, or 10 mM EDTA. The reaction buffer contained 25 mM Tris pH 7.5, 100 mM NaCl, and 0.1% DDM. Reactions were incubated for 30 min at 25 °C. (D) Spot plate assay to test the complementation of BW25113 Δ*pgpB* Δ*ybjG* Δ*lpp*::kan sensitivity to EDTA. Cells were transformed with the indicated plasmids encoding IPTG-inducible His-UppP (H-UppP), UppP, PgpB-his (PgpB-h), YgjG, or LpxT or control plasmids encoding mCherry or mKO. Plasmids encoding PgpB-his and YbjG complemented the sensitivity to EDTA with or without IPTG, though in the case of YbjG, overexpression caused slight toxicity; hence, the complementation worked better without IPTG. Plasmids encoding LpxT, UppP, or His-UppP failed to complement sensitivity to EDTA. Cells were plated in LB-Agar medium with the indicated additives and incubated for 40 h at 37 °C before imaging. EDTA was added at 2 mM and IPTG at 0.1 mM. Images shown. Three colonies from each strain were tested twice; representative images are shown.

To examine the functional differences between UppP and PgpB phosphatases, we probed the influence of divalent cations on the activities of both enzymes. To this end, we performed our MS assay for phosphatase functions of PgpB in the presence of EDTA. We find that the activities of *E. coli* and *B. subtilis* PgpB homologs are unaffected by EDTA (Figures 3A and S6). In contrast, UppP binds the substrate C_55_-PP in the presence of EDTA with little to no evidence of dephosphorylation (Figure 3B). This observation indicates the essential nature of the metal cations in this reaction. We surmise that divalent cations (Mg^2+^ and/or Ca^2+^) carried over during purification are sufficient to drive these activities in MS assays.

We next performed orthogonal thin-layer chromatography (TLC) analyses of protein/substrate mixtures with and without CaCl_2_ and EDTA. The data showed that the activity of PgpB^Ec^ is unaffected by CaCl_2_ or EDTA, but UppP activity is completely inhibited by EDTA (Figure 3C). Unlike PgpB^Ec^, the activity of UppP is further enhanced by CaCl_2_ (Figure 3C), confirming previous reports.^49,53^ We then questioned whether catalysis by other membrane PAP2-type phosphatases, apart from PgpB, is independent of divalent cations. To this end we performed a similar assay on *E. coli* YbjG (another phosphatase belonging to the PAP2-type family). The results indicated that the activity of YbjG against C_55_-PP is unaffected by EDTA or CaCl_2_ (Figure S7), confirming that divalent cations are not critical for catalysis by the PAP2-family enzymes.

To understand the different in vivo roles of UppP-type and PAP2-type phosphatases, we investigated the EDTA sensitivity of *E. coli* BW25113-derived strains with all possible combinations of genomic deletions of all *E. coli* genes encoding enzymes that can dephosphorylate C_55_-PP: *uppP*, *pgpB*, *ybjG,* and *lpxT*. First, we tested each strain for detergent sensitivity to ensure that the impact of gene deletions is not solely due to membrane leakiness. Unlike in a previous report,^54^ we find no sensitivity to detergents for any of the strains tested except for mild sensitivity to sodium dodecyl sulfate (SDS) and deoxycholate (DOC) of strains BW25113 Δ*uppP* Δ*ybjG* Δ*lpxT* (Figure S8). Importantly, our data indicate that all stains depending on UppP for C_55_-PP phosphatase activity are highly sensitive to EDTA (Figures 3D and S8). As a control, there was no change to the SDS-PAGE migration pattern of lipopolysaccharide (LPS)^55^ extracts from mutant strains relative to the wild-type (Figure S8), suggesting that the EDTA sensitivity was not caused by a major change in the cellular amount of LPS, although changes to the LPS structure such as modifications with pyrophosphate or aminoarabinose cannot be detected by this method. We thus hypothesized that the EDTA sensitivity is caused by an impaired cellular function of UppP. If this hypothesis is correct, overexpression of the PAP2-type phosphatase enzymes PgpB or YbjG, but not of UppP, should restore the resistance to EDTA. Accordingly, only BW25113 Δ*pgpB* Δ*ybjG* Δ*lpxT* with plasmids encoding the PAP2-type phosphatases, restored the resistance to EDTA (Figures 3D and S8).

### Fine-Tuning the Enzymatic Activity of UppP

**Figure 4:**
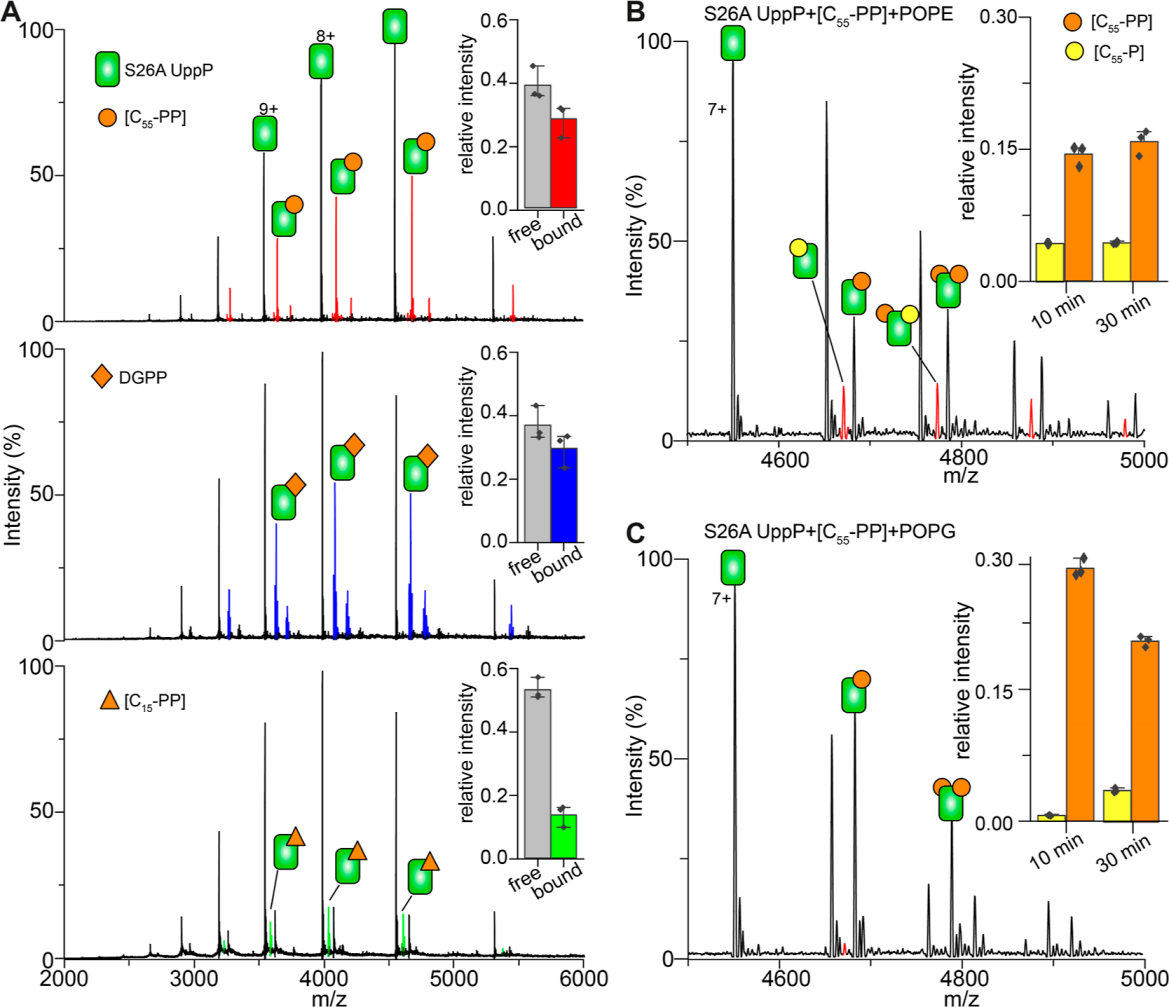
Substrate-binding affinity and effects of phospholipids on UppP activity. (A) Spectra of 3.5 μM S26A UppP titrated with 10 μM C_55_-PP, 10 μM DGPP, and 20 μM C_15_-PP. C_55_-PP and DGPP bind more favorably to the enzyme than C_15_-PP. (Inserts) mean relative abundance of ligand-free and ligand-bound S26A UppP in the spectra. Error bars are standard deviations of three replicate measurements. (B) Spectrum of 3.5 μM S26A UppP titrated with 10 μM C_55_-PP in the presence of 20 μM POPE. Protein, substrate, and lipid were incubated for 10 min prior to measurement. Peaks corresponding to product C_55_-P are highlighted. (C) Spectrum of a reaction mixture equivalent to (B) but in the presence of 20 μM POPG. The product C_55_-P is detected earlier for the assay in the presence of POPE but not in the presence of POPG. Insert, the mean relative intensity of C_55_-P and C_55_-PP bound to UppP at 10 and 30 min time points. Error bars are standard deviations of three replicate measurements.

Our data mentioned above indicate that UppP and PgpB process their substrates on the time scale of a few seconds, which makes it difficult to study the influence of cofactors, such as lipids, on enzyme function. To overcome this challenge, we decided to decrease the reaction rate by mutating the active site residues of *E. coli* UppP. We generated E21A, S26A, S27A, S26A/S27A, and R174A and S26A/R174A UppP mutants to disrupt the catalytic site.^40,49,56^ The E21 residue is predicted to activate the S27 residue, which subsequently initiates a nucleophilic attack on the terminal phosphate of C_55_-PP. However, in our MS experiments, no difference could be detected between the dephosphorylation of C_55_-PP by the UppP wild-type and the E21A mutant (Figure S9). Together with an earlier report that a double mutant E17A/E21A is inactive,^53^ this observation suggests that a glutamic acid residue E17 could also initiate the reaction. We observed only a very weak phosphatase activity in the mass spectra of the single mutants S26A or S27A even after prolonged incubation with C_55_-PP (Figures 4A and S9). This weak activity is abrogated in the case of the double mutant S26A/S27A (Figure S9), indicating that the S26 residue can partially compensate for S27 and vice versa. We also found from the mass spectra of R174A and the double mutant S26A/R174A that these mutants are inactive with respect to the dephosphorylation of C_55_-PP (Figure S9). We, therefore, conclude that R174 is critical in generating the phosphoenzyme intermediate in accordance with previous reports.^40,56^

We next leveraged the slow activity of the UppP S26A mutant to compare the binding affinity of UppP to different substrates. To this end, we recorded native MS spectra for S26A UppP incubated with C_55_-PP, DGPP or C_15_-PP. Spectra were recorded after 10 min of equilibration to mitigate the loss of binding due to slow processing of C_55_-PP by this mutant UppP. Comparing mass spectra under identical conditions, we find that while UppP binds C_55_-PP and DGPP to comparable extents, it does bind more favorably to the “true” carrier lipid substrate C_55_-PP than to C_15_-PP by a factor of 2 (Figure 4A).

We next assessed the role of phospholipids on UppP function by comparing affinity for C_55_-PP in the presence of 20 μM 1-palmitoyl-2-oleoyl-*sn*-glycero-3-phosphoethanolamine (POPE) and 20 μM 1-palmitoyl-2-oleoyl-*sn*-glycero-3-phosphoglycerol (POPG) (Figure 4B,C). We selected POPE and POPG as these represent the two most abundant lipid classes in the *E. coli* membrane based on headgroup chemistry.^57^ Mass spectra recorded for the mixture of S26A UppP with C_55_-PP and POPE reveal product formation within 10 min of mixing; this is a faster time scale compared to ∼30 min for the reaction performed without adding phospholipids (cf. Figure S9). Accordingly, we detect a lower extent of C_55_-PP binding to UppP in the presence of POPE, than in the presence of POPG on the same time scale, which is compensated for by the correspondingly higher amount of C_55_-P (Figure 4B,C). By contrast, neither the reaction kinetics nor the extent of C_55_ PP binding is impacted by the presence of POPG (Figure 4C). We, therefore, conclude that UppP activity is enhanced by POPE, in part explaining its affinity for endogenous PE (Figure 1C).

### In Situ Inhibition of UppP and PgpB by Antibiotics

**Figure 5:**
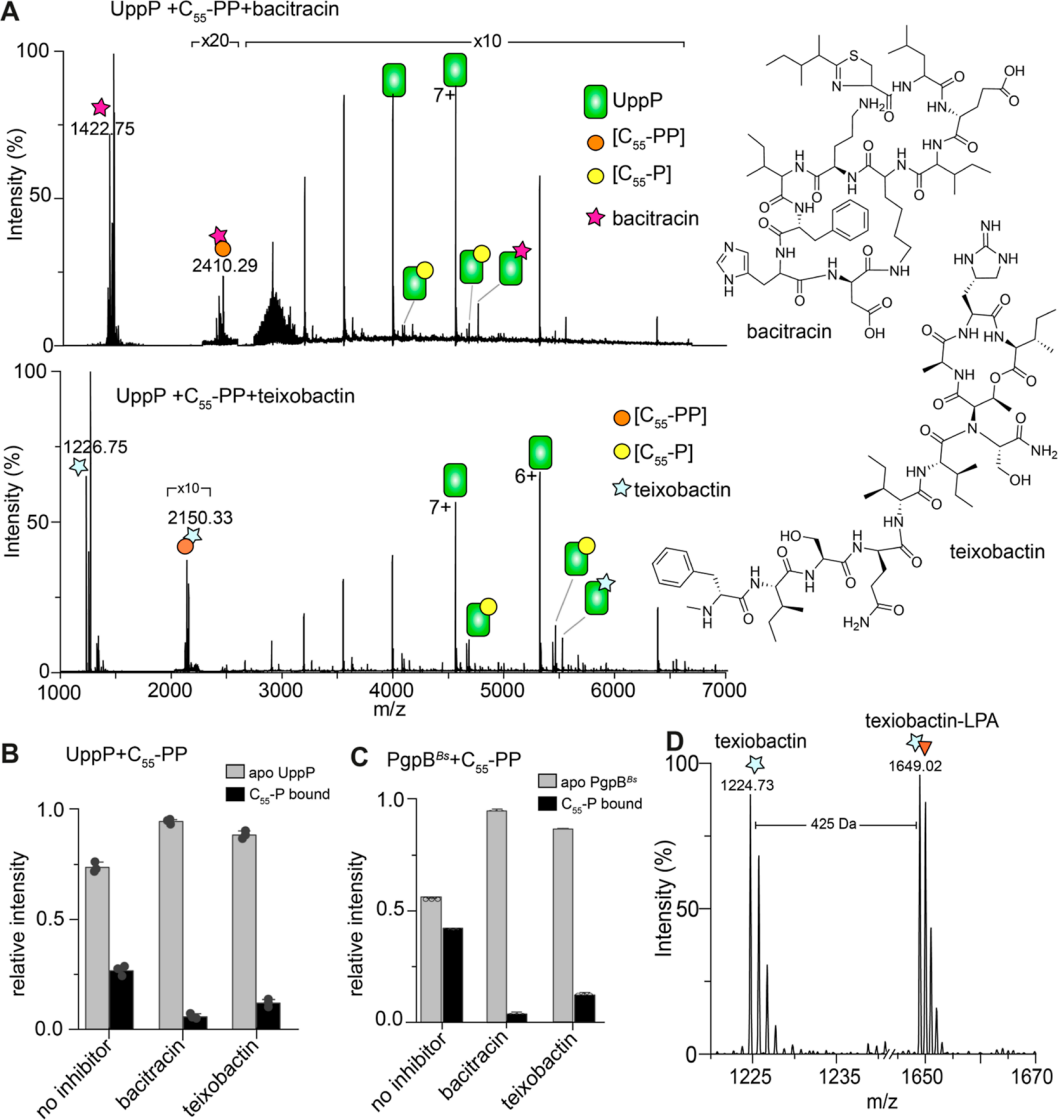
Inhibition of UppP and PgpB activities by antibiotics. (A) Spectra for a solution containing UppP (3.5 μM) and 20 μM C_55_-PP incubated in the presence of 100 μM bacitracin (top) and 100 μM teixobactin (lower panel) for 30 min. Only a small amount of enzyme–product complex is detected in the presence of both inhibitors, reflecting that both inhibitors sequester the lipid substrate C_55_-PP from the enzyme. The *m*/*z* values for antibiotics and their complexes with the substrate are labeled. Shown on the right are the chemical structures of bacitracin and teixobactin. (B,C) The relative intensities of enzyme–product complexes in the spectra for UppP- and PgpB-mediated formation of C_55_-PP. (D) The low *m*/*z* region of spectrum acquired for PgpB/LPA mixture in the negative electrospray ionization mode. Teixobactin form a stable complex with LPA.

The ability to monitor the enzymatic activities of UppP and PgpB in real-time suggests that these activities could be inhibited in situ by adding specific antibiotics that sequester substrates from these enzymes. We selected an established antibiotic, bacitracin, and a newly identified antibiotic, teixobactin—both of which are known to sequester C_55_-PP. First, we tested the impact of bacitracin on the dephosphorylation of C_55_-PP and DGPP by UppP. We incubated 100 μM of bacitracin with 3.5 μM UppP, added 10 μM of each substrate, and acquired spectra after 30 min of equilibration. Compared to the uninhibited reactions, we detect little or no protein-bound substrate or product molecules (Figures 5A,B and S10). We attribute the low amount of detected product to the presence of bacitracin in solution, which outcompetes the protein for substrate binding. Accordingly, we observe a metal-mediated complex of bacitracin with a substrate molecule in the lower *m*/*z* region of the spectra. Although bacitracin is present in the reaction mixture at >28-fold molar excess of UppP, residual activities still occur because the complex (bacitracin-metal ion-C_55_-PP) exist in a state of dynamic equilibrium with the free C_55_-PP molecules, some of which are still processed by the enzyme. Therefore, the enzymatic activity of UppP is halted by bacitracin because of reduced availability of substrate and not necessarily by enzyme deactivation, in accord with its established MoA.^58^

We next studied the effect of teixobactin on phosphatase activities. Recent structural data suggest that teixobactin forms a 1:1 complex with lipid II by binding specifically to the pyrophosphate-sugar moiety of lipid II, accounting for the lack of teixobactin resistance in Gram-positive bacteria.^59^ Considering its mode of recognition, it was also shown to inhibit C_55_-PP processing by YbjG.^7,59,60^ To understand whether the same is true for other phosphatases, we incubated solutions containing 3.5 μM UppP and 10 μM C_55_-PP with 100 μM teixobactin and recorded spectra after 30 min of incubation. The results show that analogous to bacitracin, teixobactin does not bind to UppP but sequesters the substrate C_55_-PP, leaving the enzyme predominantly in its apo form (Figure 5A,B).

Similarly, bacitracin and teixobactin sequester substrate C_55_-PP from *B. subtilis* PgpB (Figure 5C). However, the relative intensities of product detected for inhibition with bacitracin are generally lower than those with teixobactin. This data indicated that bacitracin is more effective in the sequestration of C_55_-PP compared to teixobactin (Figure 5B,C). Teixobactin coordinates with other cell wall precursors, including lipid II, this might enhance its efficacy overall and mitigate resistance development.^7^ We asked whether teixobactin coordinates with other cellular targets, such as LPA. Indeed, incubation of teixobactin with LPA produced a spectrum with peaks corresponding to teixobactin in complex with LPA (Figure 5D). By contrast, bacitracin did not form a complex with LPA, suggesting that bacitracin lacks stable binding with monophosphate-containing lipids. Together this data suggests that bacitracin and teixobactin can coordinate with other physiological lipids containing terminal phosphates.

Finally, we performed cell viability assays to evaluate the impact of bacitracin on various *E. coli* strains that are deficient in one or more phosphatase genes (Figure S11). We included strains lacking LpxT as this phosphotransferase can (marginally) contribute to C_55_-PP recycling.^61^ Normally, *E. coli* would be insensitive to bacitracin, as this molecule is too big to cross the outer membrane. To overcome this, we rendered the cells permeable to bacitracin by expressing the plug-less outer-membrane transporter FhuA Δ322–355 (Figure S11).^62^ Our data show that strains depending on UppP are consistently more sensitive to bacitracin than the wild type (WT). However, with PAP2 enzymes, sensitivity to bacitracin depended on the presence or absence of LpxT. Cells depending on PgpB were only as resistant to bacitracin as the WT if LpxT was present. This could indicate that either LpxT activity producing C_55_-P, as a byproduct of phosphorylation of LPS, can contribute to recycling, compensating for the absence of UppP or YbjG. Alternatively, or in addition, the phosphorylation of LPS by LpxT could help cells to resist bacitracin, for example, by stabilizing the outer membrane. Cells dependent on YbjG were only as resistant to bacitracin as the WT if LpxT was absent, suggesting that LpxT hindered YbjG function. While the relative contribution of UppP and the PAP2 enzymes to carrier lipid recycling is complex, our results suggest that different phosphatases have evolved to rescue the cell in the face of environmental assaults, for example, exposure to antibiotics or metal ion chelators. Overall, these observations indicate that bacitracin can impact the cellular functions of both UppP and PgpB.

## Conclusions

In our study, we investigated the activities of *E. coli* integral membrane phosphatases using native MS and cell-based assays. We report a previously unknown lipid-mediated dimerization of UppP and highlight the divergent lipid and divalent cation requirements for the UppP-type and PAP2-type membrane phosphatases. Systematically, we showed how the enzymatic activity of UppP can be controlled by both mutational analysis and the addition of phospholipids. Our data further reflect the substrate selectivity of *E. coli* UppP for the peptidoglycan pathway by mediating the recycling of lipid carriers, while the *E. coli* PAP2-type phosphatase PgpB preferentially processes the phospholipid precursor substrate. Furthermore, our data highlight that the activity of PgpB, unlike UppP, is insensitive to the metal chelator EDTA or CaCl_2_, indicating that PgpB does not use divalent cations for catalysis. Accordingly, we find that *E. coli* strains that depend on UppP, but not PgpB, for phosphatase activity, are highly sensitive to EDTA. Our results, therefore, indicate that the evolution of different phosphatase families in *E. coli* could be necessitated by the need to adapt to environments with different availability of divalent cations.

Previous studies have used native MS to monitor substrate binding and catalysis by membrane proteins.^18,19^ Here we demonstrate that native MS can report changes to the enzyme–substrate and enzyme–product complexes of membrane phosphatase proteins in real time. Information regarding the nature and stoichiometry of enzyme–substrate or enzyme-intermediate complexes is often lost with conventional monitoring of substrate consumption and product formation after quenching the reactions. Thus, with improved time resolution, our approach can potentially be used to detect even short-lived intermediates, to aid in elucidating reaction mechanisms. The ability to monitor enzymatic activities in real-time using native MS has allowed us to directly investigate the effects of antibiotics. By shedding light on the specific features of each enzyme that catalyzes related biochemical processes, our approach will foster an understanding of drug resistance mechanisms and aid drug discovery.

Sequestration of peptidoglycan precursors by peptide antibiotics such as nisin,^63^ bacitracin^58^ and teixobactin^59,60,64^ has been proposed. This study provides direct evidence that these antibiotics can directly outcompete the relevant membrane enzymes for substrates, thus, confirming the proposed mechanisms. We further show that apart from the lipid carrier, bacitracin can complex with DGPP, another diphosphate-containing lipid. Similarly, teixobactin can potentially interact with physiological lipids such as LPA that have a terminal phosphate group. The relative contributions of different potential lipid substrates to the cellular mode of action of teixobactin require further investigation.

More generally, this study highlights the possibility of using native MS as a platform for testing derivatives of established antibiotics to ascertain whether they retain similar modes of action. On a broader scale, the ability of native MS to screen for complexes formed between membrane enzymes, substrates, and antibiotics offers a complementary approach to understanding the MoA and subsequent optimization of antibiotic candidates.

## Supporting Information Available

Experimental procedures and additional data sets, including tables containing all the strains, plasmids, oligonucleotides used and all experimentally measured masses (PDF)
