# Supplementary material for: Real-Time Biosynthetic Reaction Monitoring Informs the Mechanism of Action of Antibiotics
Source: J Am Chem Soc. 2024 Mar 1;146(10):7007–17. doi: 10.1021/jacs.4c00081 (PMC10941186; doi:10.1021/jacs.4c00081)
Supplement: Supplementary file 1 — ja4c00081_si_001.pdf [file ja4c00081_si_001.pdf]

Supporting information

Real-time biosynthetic reaction monitoring informs the mechanism of action of antibiotics

Abraham O. Oluwole[1,2], Victor M. Hernández-Rocamora[3], Yihui Cao[4], Xuechen Li[4], Waldemar Vollmer[3,5], Carol V. Robinson[1,2*], Jani R. Bolla[2,6*]

[1]Department of Chemistry, University of Oxford, South Parks Road, Oxford, OX1 3QZ, UK.

[2]The Kavli Institute for Nanoscience Discovery, University of Oxford, South Parks Road, Oxford, OX1 3QU, UK.

[3]Centre for Bacterial Cell Biology, Biosciences Institute, Newcastle University, Richardson Road, Newcastle upon Tyne, NE2 4AX, UK.

[4]Department of Chemistry, State Key Laboratory of Synthetic Chemistry, The University of Hong Kong, Pokfulam Road, Hong Kong SAR 999077, China

[5]Institute for Molecular Bioscience, University of Queensland, Carmody Road, Brisbane, Queensland 4072, Australia.

[6]Department of Biology, University of Oxford, South Parks Road, Oxford, OX1 3RB, UK.

Email: carol.robinson@chem.ox.ac.uk; jani.bolla@biology.ox.ac.uk

Table of Contents

Methods 3

Bacterial strains and growth conditions 3

Construction of *E. coli* deletion strains 3

Cloning 3

Protein expression and purification 4

UppP and PgpB 4

YbjG 5

C_{55}-PP synthesis 5

Sample Preparation for native mass spectrometry 5

Native mass spectrometry 6

Analysis of co-purified lipids 6

Thin layer chromatography (TLC) 6

Lipopolysaccharide (LPS) isolation, detection and quantification 7

Table S1: Strains used in this work. 8

Table S2: Plasmids used in this work. 9

Table S3: Oligonucleotides used in this work. 10

Supplementary Figures 12

Fig. S1: MS analyses of lipids copurified with UppP. 12

Fig S2: Lipid-stabilized UppP dimer observed in other detergents. 13

Fig. S4: Real-time monitoring of UppP interactions with C_{15}-PP or C_{55}-PP and inhibition by EDTA. 15

Fig. S5: *E. coli* PgpB processes DGPP faster and more efficiently than C_{55}-PP. 16

Fig. S6. Activity of *B. Subtilis* PgpB towards C_{55}-PP, DGPP and lysophosphatidic acid (LPA). 17

Fig. S7. C_{55}-PP phosphatase activity of YbjG is not sensitive to EDTA. 18

Fig. S8. A. Spot plate assay to test EDTA, SDS and DOC sensitivity of BW25113 *E. coli* strains with different combinations of deletions in *uppP, lpxT, pgpB and ybjG* genes. 19

Fig. S9. Native MS (7+ charge state) for different UppP mutants 19

Fig. S10: Effect of EDTA and bacitracin on UppP function. 20

Fig. S11: Cells depending on a single phosphatase have different bacitracin sensitivity 21

Methods

Bacterial strains and growth conditions

Strains used in this study are listed in Table S1. Bacteria were grown on LB plates or in liquid LB medium (10 g/L tryptone, 5 g/L yeast extract, 10 g/L NaCl for liquid media or 15 g/L for agar plates) at 37°C. Growth of bacteria for protein was in LB or Terrific Broth (24 g/L yeast extract, 20 g Tryptone, 0.4% glycerol, 17 mM KH_2PO_4, 72 mM K_2HPO_4), containing antibiotic for selection. Antibiotics were used at the following concentrations: Chloramphenicol (Cam, 25 µg/mL); Kanamycin (Kan, 50 µg/mL), and Ampicillin (Amp, 100 µg/mL).

Construction of *E. coli* deletion strains

Deletion strains were obtained by moving *kan*-marked alleles from the Keio *E. coli* single gene knockout library[1] by P1 phage transduction[2] or by recombineering using the pKD46 and Kan cassette amplified from pKD4 as indicated in refs. [1, 3] Afterward, the *kan* cassette was removed by pCP20-encoded Flp recombinase to generate unmarked deletions with a FRT-site scar sequence[3]. The removal of the *kan* gene was verified by colony PCR. Strains with multiple deletions were generated by sequential P1 transduction or recombineering and *kan* cassette removal.

Cloning

All template plasmids are listed on Table S2 and oligonucleotide sequences are listed on Table S3. pET28a-*ybjG* plasmid, encoding for YbjG with an N-terminal His-tag, was amplified by PCR from genomic DNA of *E. coli* BW25113 using oligonucleotides YbjG_FW_NdeI and YbjG_REV_XhoI, and cloned into pET28a(+) with the appropriate restriction enzymes. pVMH23 was generated by two PCR fragments: an amplified product obtained from pHK226 and the oligonucleotides FhuA2pBB_frag_FW and FhuA2pBB_frag_REV (Table S2), and an amplified product obtained from pBB013 and the oligonucleotides FhuA2pBB_vect_FW and FhuA2pBB_vect_REV. The same volumes of each PCR fragment were mixed, heated to 98˚C and cooled down to room temperature. The DNA mixed was digested with DpnI and transformed into DH5α competent cells. pVMH9 was obtained by the same procedure, using pET28a-*uppP* and the oligonucleotides his*uppP*_frag_FW and his*uppP*_frag_REV, and pBB012 and oligonucleotides his*uppP*_pBBvect_fw and his*uppP*_pBBvect_rev. pVMH10 was obtained by the same procedure using pET28a-pgpB and the oligonucleotides *pgpB*his_frag_FW and *pgpB*his_frag_REV, and pBB013 and oligonucleotides *pgpB*his_pBBvect_fw and *pgpB*his_pBBvect_rev. pVMH13 was obtained by the same procedure using BW25113 genomic DNA and the oligonucleotides *ybjG*_frag_FW and *ybjG*_frag_REV, and pBB013 and oligonucleotides *ybjG* _pBBvect_fw and *ybjG*_pBBvect_rev. pVMH16 was obtained by the same procedure using BW25113 genomic DNA and the oligonucleotides *lpxT*_frag_FW and *lpxT*_frag_REV, and pBB013 and oligonucleotides *lpxT*_pBBvect_fw and *lpxT*_pBBvect_rev. pVMH11 was obtained by removal of the encodes His-tag in plasmid pVMH9 with primers *uppP*_deltaHis_FW and *uppP* _deltaHis_REV using Q5® Site-Directed Mutagenesis Kit (New England Biolabs). A codon-optimized gene block for *Bacillus subtilis* PgpB (Integrated DNA Technologies) was cloned into pET28a vector between the restriction

sites BamHI and XhoI using Infusion cloning kit (Takara) according to the manufacturer's protocol. All constructs were confirmed by DNA sequencing of the genes of interest.

Protein expression and purification

UppP and PgpB

Plasmids encoding *E. coli* PgpB and UppP (ref.[4]) were used to transform chemically competent *E. coli* C43(DE3). Plasmid encoding *Bacillus subtilis* was used to transform chemically competent *E. coli* C41(DE3). Single colonies were used to inoculate LB media containing kanamycin and were grown overnight at 37°C. 10 mL of the overnight culture were used to inoculate 1L of LB containing Kanamycin and were grown to OD_{600nm} of 0.6-0.8 after which temperature was lowered to 18°C. Protein expression was induced by adding IPTG at 0.5 mM and cells were harvested after 20 hrs by centrifugation at 5000 *g* for 10 min. All subsequent steps were performed at 4°C. Cell pellets are homogenised in lysis buffer (50 mM Tris-HCL, 150 mM NaCl, pH8.0) supplemented with EDTA-free protease inhibitor cocktail tablets (Roche), 5 mM β-mercaptoethanol, 0.2 mg mL^{-1} of deoxyribonuclease I, and 5 mM $MgCl_2$. Cells were lysed by 3-4 passes through a microfluidizer (Microfluidics) at 20,000 psi and unbroken cells were pelleted from the lysates by centrifugation at 20,000 *g* for 20 min. The membrane fraction was separated from the clarified lysates by centrifugation at 100,000 *g* for 90 min. Membranes were homogenised in a buffer containing 20% glycerol, 20 mmol dm^{-3} Tris-HCL, 300 mM NaCl, pH 8.0. Aliquots of membrane corresponding to the harvest from 4 L cell culture were solubilised immediately or flash frozen in liquid nitrogen and stored at -80°C until needed.

Membrane fractions were solubilised by adding DDM at a final concentration of 2% and incubation for 2 h. Gentle solubilisation of *E. coli* PgpB was performed by solubilising the membrane preparation (harvest of 4L cell culture) with 0.5% DDM for 1 hr. Nonsolubilised materials were removed by centrifugation at 20,000 *g* for 20 min and imidazole was added to the recovered supernatant at a final concentration of 20 mM. The sample was passed over 5-mL HisTrap HP column pre-equilibrated with buffer B20 (20 mM imidazole, 0.03% DDM, 10% glycerol, 20 mM Tris-HCL, 150 mM NaCl, pH 8.0). The column was washed with 100 mL of buffer B50 (50 mM imidazole, 0.03% DDM, 10% glycerol, 20 mM Tris-HCL, 150 mM NaCl, pH 8.0) and 50 mL of buffer B80 (80 mM imidazole, 0.03% DDM, 10% glycerol, 20 mM Tris-HCL, 150 mM NaCl, pH 8.0). To achieve delipidation of UppP, i.e., reducing the amount of copurified phospholipids, the membrane was solubilised with 2% DDM for 16 h and after binding to the column, the protein was additionally washed with 200 mL of B50 supplemented with 0.5% DDM. Bound proteins were eluted from the column using buffer B300 (300 mM imidazole, 0.03% DDM, 10% glycerol, 20 mmol dm^{-3} Tris-HCL, 300 mM NaCl, pH 8.0). Fractions containing pure proteins were pooled and concentrated using an Amicon concentrator device with a nominal molecular weight cut-off of 100 kDa. Proteins were finally loaded onto a Superdex S200 Increase column pre-equilibrated with a buffer containing 0.03% DDM, 10% glycerol, 20 mM Tris-HCL, 300 mM NaCl, pH 8.0. Fractions were pooled, concentrated as previously described, aliquoted and snap-frozen in liquid nitrogen, and stored at -80°C until needed.

YbjG

C43(DE3) cells freshly transformed with pET28a-ybjG were grown in 2 L terrific broth (24 g/L yeast extract, 20 g Tryptone, 0.4% glycerol, 17 mM KH_2PO_4, 72 mM K_2HPO_4) at 37°C to an OD_{600} of 0.6, then 1 mM IPTG was added to induce expression and cells were incubated at 20°C for 16 h. Cells were pelleted by centrifugation at 7000×g for 10 min and resuspended in 80 mL of buffer A (50 mM Tris-HCl, 1 M NaCl, 10% glycerol, at pH 7.5) supplemented with DNAase I, protease inhibitor cocktail (1:1000 dilution), 2 mM PMSF. Cells were disrupted by sonication on ice and centrifuged (130000×g for 1 h at 4°C) to pellet the membrane fraction. The pellet was resuspended in buffer B (25 mM Tris-HCl, 500 mM NaCl, 1 mM $MgCl_2$, 2% DDM, at pH 7.5) by stirring at 4°C for 16 h. The extracted membraned was separated from debris by centrifugation (130000×g for 1 h at 4°C) and incubated for 1 h with 4 mL Ni^{2+}-NTA beads (Novagen) at 4°C equilibrated in buffer C (25 mM Tris pH 7.5, 500 mM NaCl, 1 mM $MgCl_2$, 0.05% DDM). Beads were washed 10 times with buffer C supplemented with 40 mM imidazole and the protein was eluted with 3 mL buffer C supplemented with 500 mM imidazole. The eluted protein was analysed by SDS-PAGE and the purest fractions were pooled and extensively dialysed against buffer D (25 mM Tris pH 7.5, 300 mM NaCl, 10% glycerol). The protein was finally concentrated using filter concentrators with a 10000 MWCO cut-off, the concentration was measured with a BCA protein concentration kit (Thermo), aliquoted and stored at -80°C.

C_{55}-PP synthesis

C_{55}-PP synthesis reactions were carried out as described previously[4] with minor modifications. Reactions (600 µL) contained 100 nmol C_{15}-PP, 1000 nmol C_5-PP and 10 nmol of UppS in 50 mM Tris-HCl pH 7.5, 50 mM KCl, and 0.05% DDM, LDAO or Triton X-100 as indicated. After overnight incubation at 37°C, the produced C_{55}-PP was extracted 3 times with 500 µL butanol/pyridine acetate at pH 4.2 (2:1). The organic phase was separated each time, combined and finally aliquoted and dried using a speed-vac concentrator. Completion of the reaction was verified by analysing the product by thin layer chromatography (TLC) and quantification of product concentration by colorimetric phosphate assay after complete digestion with UppP: reactions containing 0.5 µM UppP and ~20 µM C_{55}-PP were incubated for 2 h at 37 °C and stopped by the addition of the acidic reagent in the PiColorLock phosphate detection kit (Expedeon, UK). The amount of phosphate produced was calculated from the absorbance at 620 nm measured using a plate reader, by comparing it with a P_i standard.

Sample Preparation for native mass spectrometry

POPE and POPG (Avanti) were dissolved in chloroform: methanol (2:1 v/v) mixture and the organic solvents were removed by evaporation. 1-Octadecyl lysophosphatidic acid and 1-oleoyl lysophosphatidic (Cayman) was dissolved at 1 mg/mL in mM in 200 mM ammonium acetate (pH 8.0) and 0.05% LDAO by vertexing and then sonication in a water bath for 30 min. C_{15}-PP and C_{55}-PP solution in methanol/ammonia was dried in a SpeedVac and then resuspended in 200 mM ammonium acetate (pH 8.0) and 0.05% LDAO to a final concentration of 1 mM. The stock solution of bacitracin (Sigma-Aldrich) was prepared by dissolving the powder at 1 mM in 200 mM ammonium acetate (pH 8.0) and 0.05% LDAO. Teixobactin was synthesized according to the published procedure,[5] dissolved

in DMSO at a concentration of 10 mM, and subsequently diluted to 1 mM with 200 mM ammonium acetate (pH 8.0) and 0.05% LDAO. Protein was thawed on ice and buffer-exchanged into 200 mM ammonium acetate (pH 8.0) and 0.05% LDAO using a centrifugal buffer exchange device (Micro Bio-Spin 6, Bio-Rad).

Native mass spectrometry

About 3 µL of a protein aliquot was transferred into a gold-coated borosilicate capillary (Harvard Apparatus). The capillary was mounted on the nano ESI source of a Q-Exactive hybrid quadrupole-Orbitrap mass spectrometer (Thermo Fisher Scientific, Bremen, Germany). For kinetic experiments, the instrument parameters were pre-optimised via test measurements prior to mixing protein with the desired substrate to minimise the lag times. For measurements longer than 2 minutes, fresh aliquots for the same sample vail were loaded. The instrument settings were 1.2 kV capillary voltage, S-lens RF 200%, argon UHV pressure 3.3×10^{-10} mbar, capillary temperature 100 °C, and resolution of the instrument was set to 17,500 at a transient time of 64 ms. Voltages of the ion transfer optics –injection flatapole, inter-flatapole lens, bent flatapole, and transfer multipole were set to 5, 3, 2, and 30 V respectively. The noise level was set at 3. Unless otherwise stated, proteins were activated by applying 100 V in the high-energy collisional dissociation cell without in-source trapping. Data visualised and exported for processing using the Qual browser of Xcalibur 4.1.31.9 (Thermo Scientific). Spectral deconvolution was performed using UniDec.[6] Typically the m/z range was set at 2000-10000 without baseline subtraction with the charge range set at 1-20, the mass range was set at 20000-70000 Da, and masses were sampled every 1.0 Da with the peak detection threshold minimum of 5%. Relative binding affinities were obtained from deconvoluted spectra by dividing the intensity of ligand-bound protein peaks by the sum of the intensities of ligand-bound and ligand-free protein peaks. All measurements were performed at least three times and yielded similar results.

Analysis of co-purified lipids

Lipids copurified with UppP were extracted by adding 100 µL chloroform: methanol (2:1 vol/vol) mixture to 50 µL protein in 200 mM ammonium acetate, pH8, 0.05% LDAO. The mixture was centrifuged at 20,000 g for 10 min to separate the organic and aqueous phases. The organic phase containing copurified lipids was dried under vacuum at 45°C for 30 min. The resulting lipid film was resuspended in a buffer containing 10 mM ammonium formate, 0.1 %(vol/vol) formic acid, and 60 %(vol/vol) acetonitrile. The sample was analysed in the negative ESI polarity on an Eclipse Tribrid mass spectrometer (Thermo Fisher Scientific) using a CID activation of 175 V for MS^1 and an additional 28-32 V for MS^2.

Thin layer chromatography (TLC)

Reactions with UppP and PgpB (40 µL) contained 1 µM of enzyme, 35 µM C_{55}-PP in 50 mM Tris-HCl pH 7.5, 100 mM NaCl, and 0.1% of DDM. When indicated, 10 mM $CaCl_2$ or 10 mM EDTA were present and pre-incubated with the enzymes before the addition of substrate. Reactions were incubated at 25 °C for 30 min. and stopped by the addition of 50 µL butanol/pyridine acetate pH 4.2 (2:1). Reactions

with YbjG (40 µL) contained 0.05 mg/mL of semi-purified enzyme, 31.2 µM C_{55}-PP in 50 mM Tris-HCl pH 7.5, 150 mM NaCl, and 0.2% DDM. When indicated,15 mM EDTA, 0.1% Triton X-100 or 0.1% LDAO were added. Reactions were incubated at 25 °C for 30 min and stopped as indicated above for PgpB and UppP reactions. After the addition of butanol/pyridine acetate, the organic phase was separated and spotted on a HPTLC alumina silica gel 60 plate (Merck). TLC was performed using chloroform-water-ammonia (88:48:10:1) as the mobile phase.[7] TLC bands were stained with iodine, scanned, and analysed using ImageJ software.[8]

Lipopolysaccharide (LPS) isolation, detection and quantification

The protocol for LPS isolation was adapted from Crawford *et al*.[9]. *E. coli* strains of interest were grown in LB (supplemented with antibiotics when required) at 37°C by orbital shaking up to an optical density at 600 nm of 1.0. Cell growth was stopped by incubating flasks on ice for 10 min. Cell suspensions (2.5 ml) were pelleted by centrifugation (17000×g, 4°C, 5 min) and resuspended in 1 ml of cold PBS. Cells were pelleted and washed in PBS twice more, then pellets were resuspended in 100 µl of 2× SDS-PAGE loading buffer (ThermoFisher) and boiled at 100°C for 10 min. Samples were supplemented with 100 µl of Proteinase K (Sigma-Aldrich, 2 mg/ml stock solution in 10 mM Tris pH 6.8, 20 mM $CaCl_2$, 50% glycerol) and incubated at 65°C for 3 h with vigorous shaking (900 rpm), then boiled again.

LPS was detected by loading 10 µl of sample on Tris-Tricine gels containing 18% polyacrylamide and staining with Pro-QTM Emerald 300 Lipopolysaccharide Gel Stain Kit (ThermoFisher), according to the manufacturer’s protocol. Briefly, LPS was fixed on polyacrylamide gels by incubating twice in 50% methanol, 5% acetic acid for 45 min, then washed in 3% acetic acid twice. Carbohydrate groups from LPS were oxidised by incubating the gel with periodic acid for 30 min and washing again in 3% acetic acid three times. Gels were stained with Pro-Q Emerald 300 for 2 h in the dark and washed with 3% acetic acid twice more. LPS was visualized in a Gel Doc XR+ System (Bio-Rad) under UV light at 300 nm.

Table S1: Strains used in this work.

Strain	Relevant features, characteristics	Reference/Source
DH5alpha	Cloning strain	Thermo
BL21(DE3)	Overexpression of UppS	Sigma
C43(DE3)	Overexpression of UppP, PgpB and YbjG	Sigma
BW25113	*lacI^q^* *rrnB*$_{T14}$ Δ*lacZ*$_{WJ16}$ *hsdR514* Δ*araBAD*$_{AH33}$ Δ*rhaBAD*$_{LD78}$	[3]
BW25113 Δ*lpp*::kan	BW25113 Δ*lpp*::kan	(This work)
BW25113 Δ*pgpB*::kan	BW25113 Δ*pgpB*::kan	(This work)
BW25113 Δ*uppP*::kan	BW25113 Δ*uppP*::kan	(This work)
BW25113 Δ*ybjG*::kan	BW25113 Δ*ybjG*::kan	(This work)
BW25113 Δ*lpxT*::kan	BW25113 Δ*lpxT*::kan	(This work)
BW25113 Δ*pgpB* Δ*lpxT*::kan	BW25113 Δ*pgpB*::frt Δ*lpxT*::kan	(This work)
BW25113 Δ*uppP* Δ*lpxT*::kan	BW25113 Δ*uppP*::frt Δ*lpxT*::kan	(This work)
BW25113 Δ*ybjG* Δ*lpxT*::kan	BW25113 Δ*ybjG*::frt Δ*lpxT*::kan	(This work)
BW25113 Δ*pgpB* Δ*ybjG*::kan	BW25113 Δ*pgpB*::frt Δ*ybjG*::kan	(This work)
BW25113 Δ*pgpB* Δ*ybjG* Δ*lpxT*::kan	BW25113 Δ*pgpB*::frt Δ*ybjG*::frt Δ*lpxT*::kan	(This work)
BW25113 Δ*uppP* Δ*ybjG*::kan	BW25113 Δ*uppP*::frt Δ*ybjG*::kan	(This work)
BW25113 Δ*uppP* Δ*ybjG* Δ*lpxT*::kan	BW25113 Δ*uppP*::frt Δ*ybjG*::frt Δ*lpxT*::kan	(This work)
BW25113 Δ*uppP* Δ*pgpB::*kan	BW25113 Δ*uppP*::frt Δ*pgpB::*kan	(This work)
BW25113 Δ*uppP* Δ*pgpB* Δ*lpxT*::kan	BW25113 Δ*uppP*::frt Δ*pgpB*::frt Δ*lpxT*::kan	(This work)

Table S2: Plasmids used in this work.

Plasmid	Relevant features, characteristics	Reference/Source
pKD46	Expression of λ-Red recombination system under control of arabinose-dependent promoter. Amp^R.	[3]
pKD4	Template plasmid for Kan cassette amplification. Kan^R and Amp^R.	[3]
pCP20	FLP expression, temperature sensitive replication; Cam^R and Amp^R.	[3]
pBB012	pBR322 origin, *lacI*, mCherry under control of pTrcHis promoter (IPTG-inducible), Amp^R.	(gift from Tanneke den Blaauwen)
pBB013	P15A origin, *lacI*, mKO under control of pTrcHis promoter (IPTG-inducible), Cm^R.	(gift from Tanneke den Blaauwen)
pVMH9	His-UppP sequence substituting mCherry in pBB012, Amp^R.	(this work)
pVMH10	*pgpB* sequence substituting mKO in pBB013, Cm^R.	(this work)
pVMH11	*uppP* sequence substituting mKO in pBB013, Cm^R.	(this work)
pVMH13	*ybjG* sequence substituting mKO in pBB013, Cm^R.	
pVMH16	*lpxT* sequence substituting mKO in pBB013, Cm^R.	(this work)
pVMH23	FhuA Δ322-355 sequence substituting mCherry in pBB012, Amp^R.	(this work)
pHK226	pBluescriptSK+ derivative containing FhuA Δ322-355 under control of its chromosomal promoter	[10]
pET28a-UppP	pET28a(+) derivative, for overproduction of N-His_6-UppP, encoding for an N-terminal thrombin cleavage site	[4]
pET28a-pgpB	pET28a(+) derivative, for overproduction of PgpB-LE-His_6	[4]
pET28a-pgpB	pET28a(+) derivative, for overproduction of N-His_6-PgpB of B. subtilis, encoding for an N-terminal thrombin cleavage site	(this work)
pET28a-ybjG	pET28a(+) derivative, for overproduction of N-His_6-YbjG, encoding for an N-terminal thrombin cleavage site	(this work)
pET28a-ispU	pET28a(+) derivative, for overproduction of N-His_6-UppS, encoding for an N-terminal thrombin cleavage site	[4]

Table S3: Oligonucleotides used in this work.

Name	Sequence 5'→3'	Construct
FhuA2pBB_frag_FW	ACAGGAAACAGACCATGGCGCGTTCCAAAACTGCT	pVMH23
FhuA2pBB_frag_REV	AAGCTTGCATGCCTTTAGAAACGGAAGGTTGCG	pVMH23
FhuA2pBB_vect_FW	CGTCGTTTCTAAAGGCATGCAAGCTTG	pVMH23
FhuA2pBB_vect_REV	TTTGGAACGCGCCATGGTCTGTTTCCTGTG	pVMH23
hisuppP_frag_FW	ACAGGAAACAGACCATGGGCAGCAGCCATC	pVMH9
hisuppP_frag_REV	AAGCTTGCATGCCTTTAAAAGAACACGAC	pVMH9
hisuppP_pBBvect_fw	TCGTGTTCTTTTAAAGGCATGCAAGCTTG	pVMH9
hisuppP_pBBvect_rev	TGGCTGCTGCCCATGGTCTGTTTCCTGTG	pVMH9
pgpBhis_frag_FW	ACAGGAAACAGACCATGGCACGTTCGATTG	pVMH10
pgpBhis_frag_REV	AAGCTTGCATGCCTATCTCAGTGGTGGTG	pVMH10
pgpBhis_pBBvect_fw	ACCACCACTGAGATAGGCATGCAAGCTTG	pVMH10
pgpBhis_pBBvect_rev	ATCGAACGTGCCATGGTCTGTTTCCTGTG	pVMH10
uppP_deltaHis_FW	AGCCATATGGCTAGCGAT	pVMH11
uppP_deltaHis_FW	CATGGTCTGTTTCCTGTG	pVMH11
ybjG_frag_FW	ACAGGAAACAGACCATGCTGGAAAATTTGAATCTCT	pVMH13
ybjG_frag_REV	AAGCTTGCATGCCTATCAGTCACGCACCCAG	pVMH13
ybjG_pBBvect_fw	GGTGCGTGACTGATAGGCATGCAAGCTTG	pVMH13
ybjG_pBBvect_rev	AAATTTTCCAGCATGGTCTGTTTCCTGTG	pVMH13
lpxT_frag_FW	ACAGGAAACAGACCATGATTAAAAATTTGCCG	pVMH16
lpxT_frag_REV	AAGCTTGCATGCCTATGATGTTAATTACTGTGAG	pVMH16
lpxT_pBBvect_fw	GTAATTAACATCATAGGCATGCAAGCTTG	pVMH16
lpxT_pBBvect_rev	AAATTTTTAATCATGGTCTGTTTCCTGTG	pVMH16
YbjG_FW_NdeI	CATCATCATATGCTGGAAAATTTGAATCTC	pET28a-ybjG
YbjG_REV_XhoI	CATCATCTCGAGATCAGTCACGCACC	pET28a-ybjG

Table S4: Representative experimental masses of proteins in apo and ligand-bound forms observed masses by native MS.

	Observed masses (Da)	Assignments/ remarks
Fig. 1B-C, Fig. S1-2	31860.73±0.23	UppP wild type (monomer expected mass 31861.97 Da)
	31921.99±1.7	UppP bound to a 60-Da cation (Ni^{2+})
	32562.5±1.23	UppP bound to a phospholipid (UppP + 701.77 Da)
	64423.50±1.01	$(UppP)_2$ bound to a phospholipid
Fig. 1D	30025.57±0.53	$PgpB^{Ec}$ (expected mass 30026.27 Da)
Fig. S3	30743.46±0.72	$PgpB^{Ec}$ bound to exogenous POPE (+717.89 Da)
	31431.32±0.25	$PgpB^{Ec}$ bound to endogenous cardiolipin (+1405.44 Da)
Fig. 2A	31941.00±0.66	UppP bound to 80.17 Da phosphate group
	32562.06±0.72	UppP bound to PE (+701.83 Da)
	32788.57±0.06	UppP bound to 1x C_{55}-PP (UppP + 927.74 Da)
	33715.57±0.48	UppP bound to 2x C_{55}-PP (UppP + 1854.74 Da)
	34643.49±0.05	UppP bound to 3x C_{55}-PP (UppP + 2782.66 Da)
	32708.26±0.09	UppP bound to 1x C_{55}-P (UppP + 847.43 Da)
	33555.40±0.75	UppP bound to 2x C_{55}-P (UppP + 1694.57 Da)
	33635.79±0.52	UppP bound to 1x C_{55}-P and 1x C_{55}-PP (UppP + 1774.96 Da)
	34563.35±0.12	UppP bound to 1x C_{55}-P and 2x C_{55}-PP (UppP + 2702.52 Da)
Fig. 3A	30804.42±0.38	PgpB bound to DGPP (PgpB + 780.25 Da)
	30724.83±0.42	PgpB bound to 1x DGP (PgpB + 700.66 Da)
	31426.33±0.54	PgpB bound to 2x DGP (PgpB + 1402.16 Da)
Fig. 3B, Fig. S4	32561.76±0.42	UppP bound to endogenous PE (+701.31 Da)
	32787.44±0.37	UppP bound to 1x C_{55}-PP (UppP + 926.99 Da)
	33714.78±0.16	UppP bound to 2x C_{55}-PP (UppP + 1854.33 Da)
	34642.67±0.79	UppP bound to 3x C_{55}-PP (UppP + 2782.22 Da)
Fig. 4A, S4, S10	32242.23±0.61	UppP bound to C_{15}-PP (+ 381.50 Da)
	32161.42±0.81	UppP bound to C_{15}-P (+ 301.19 Da)
	32641.57±0.27	UppP bound to DGPP (+780.84 Da)
	32561.77±0.10	UppP bound to DGP (+701.04 Da)
Fig 4B-C	31845.08±0.11	UppP S26A (expected mass 31845.97 Da)
	32563.36±0.26	UppP S26A bound to exogenous POPE (+718.28 Da)
	32593.83±0.05	UppP S26A bound to exogenous POPG (+748.75 Da)
	32771.88±0.36	UppP S26A bound to 1x C_{55}-PP (+926.80 Da)
	32692.70±0.17	UppP S26A bound to 1x C_{55}-P (+846.90 Da)
	32625.21±0.31	UppP S26A bound to DGPP (+780.40 Da)
	32227.61±0.09	UppP S26A bound to C_{15}-PP (+381.81 Da)
Fig. 5A, Fig. S10	33282.57±0.69	UppP bound to bacitracin (+1421.84 Da)
	33087.29±0.13	UppP bound to teixobactin (+1226.56 Da)
Fig. 5B-C, Fig. S5-S6	25890.98±0.01	$PgpB^{Bs}$ (expected mass 25891.65 Da)
	26738.28±0.11	$PgpB^{Bs}$ bound to 1x C_{55}-P (+847.30 Da)
	27585.52±0.65	$PgpB^{Bs}$ bound to 2x C_{55}-P (+1694.54 Da)
	26591.93±1.01	$PgpB^{Bs}$ bound to 1x DGP (+700.95 Da)
	27292.29±0.95	$PgpB^{Bs}$ bound to 2x DGP (+1401.31 Da)
	27994.79±0.97	$PgpB^{Bs}$ bound to 3x DGP (+2103.81 Da)
	26315.14±0.79	$PgpB^{Bs}$ bound to 1x LPA (+424.16 Da)
	26740.22±0.17	$PgpB^{Bs}$ bound to 2x LPA (+849.24 Da)
Fig. S9	31803.03±0.18	UppP E21A (expected mass 31803.93 Da)
	31845.35±0.11	UppP S26A (expected mass 31845.97 Da)
	31844.95±0.09	UppP S27A (expected mass 31845.97 Da)
	31775.79±0.78	UppP R174A (expected mass 31776.86 Da)
	31802.43±0.30	UppP S26A/R174A (expected mass 31802.94 Da)
	31829.14±0.76	UppP S26A/S27A (expected mass 31829.97 Da)
	32756.44±0.39	UppP S26A/S27A bound to 1x C_{55}-PP (+927.08 Da)
	33683.67±0.19	UppP S26A/S27A bound to 2x C_{55}-PP (+1854.32 Da)
	34611.39±0.32	UppP S26A/S27A bound to 3x C_{55}-PP (+2782.04 Da)

Supplementary Figures

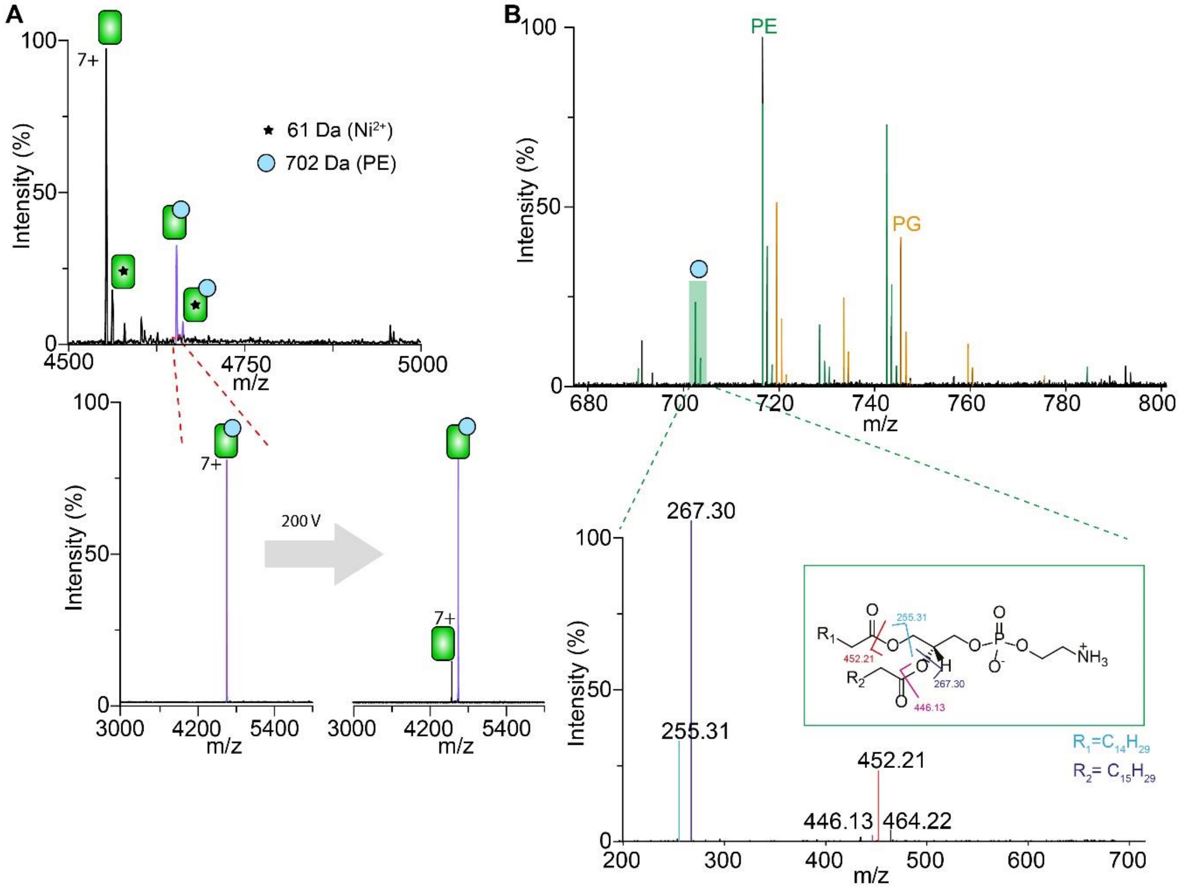

Fig. S1: MS analyses of lipids copurified with UppP. A. A lipid-bound UppP monomer state yields ligand-free UppP monomer upon collision-induced dissociation. The loss of mass 702-Da suggested the bound ligand is a phospholipid. **B**. LC-MS analysis of lipid extracts from purified UppP yielded peaks corresponding to phospholipids, including this 702-Da lipid. MS/MS on this peak resulted in a fragmentation pathway consistent with 1-hexadecanoyl-2-(heptadecenoyl)-glycerol-3-phosphoethanolamine, PE (16:0/17:1).

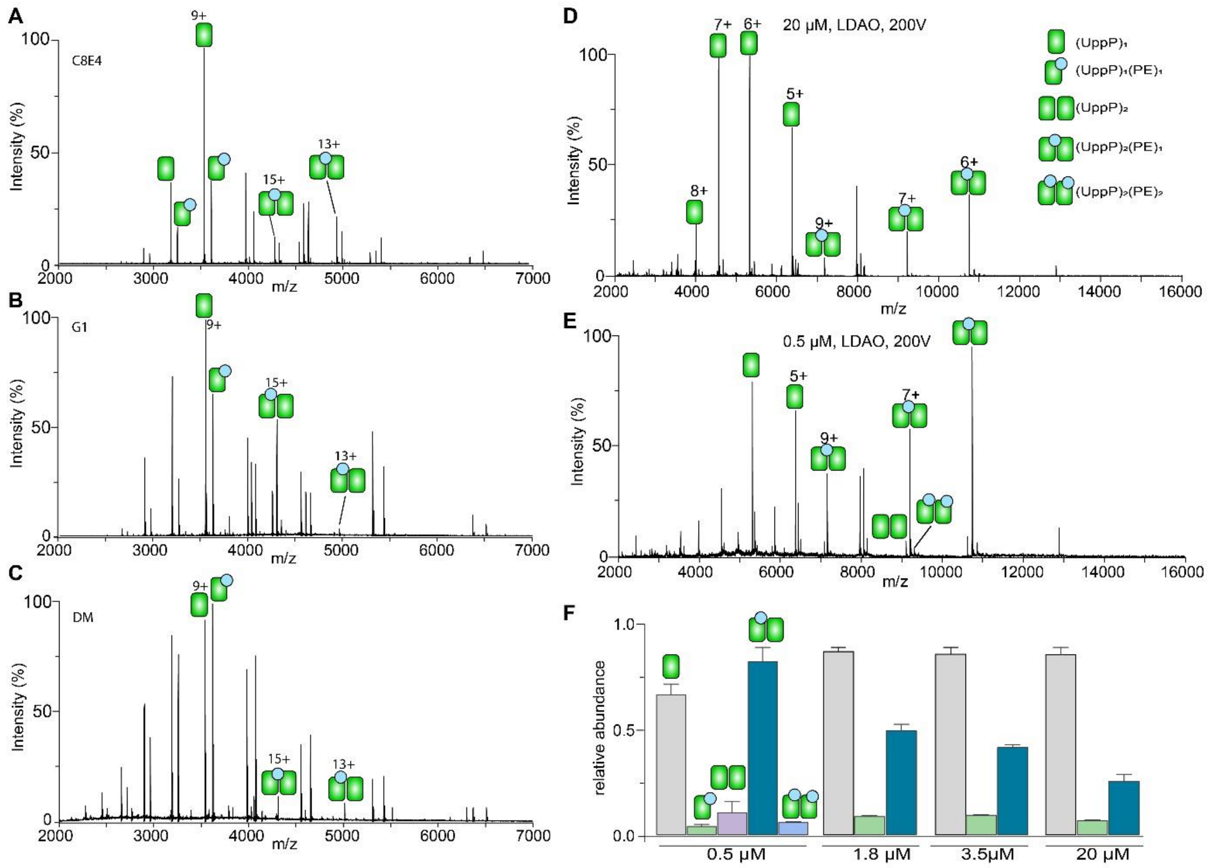

Fig S2: Lipid-stabilized UppP dimer observed in other detergents. **A-C**. Spectra recorded for UppP in C8E4, G1 (ref. [11]) and DM micelles. The mass spectrometer was optimized to maximize the transmission of dimeric species by using an activation voltage of 200V. **D,E**. Spectra of UppP released from LDAO micelles at 0.5 µM and 20 µM concentrations. **F**. Relative abundance of UppP species released from LDAO micelles at different protein concentrations.

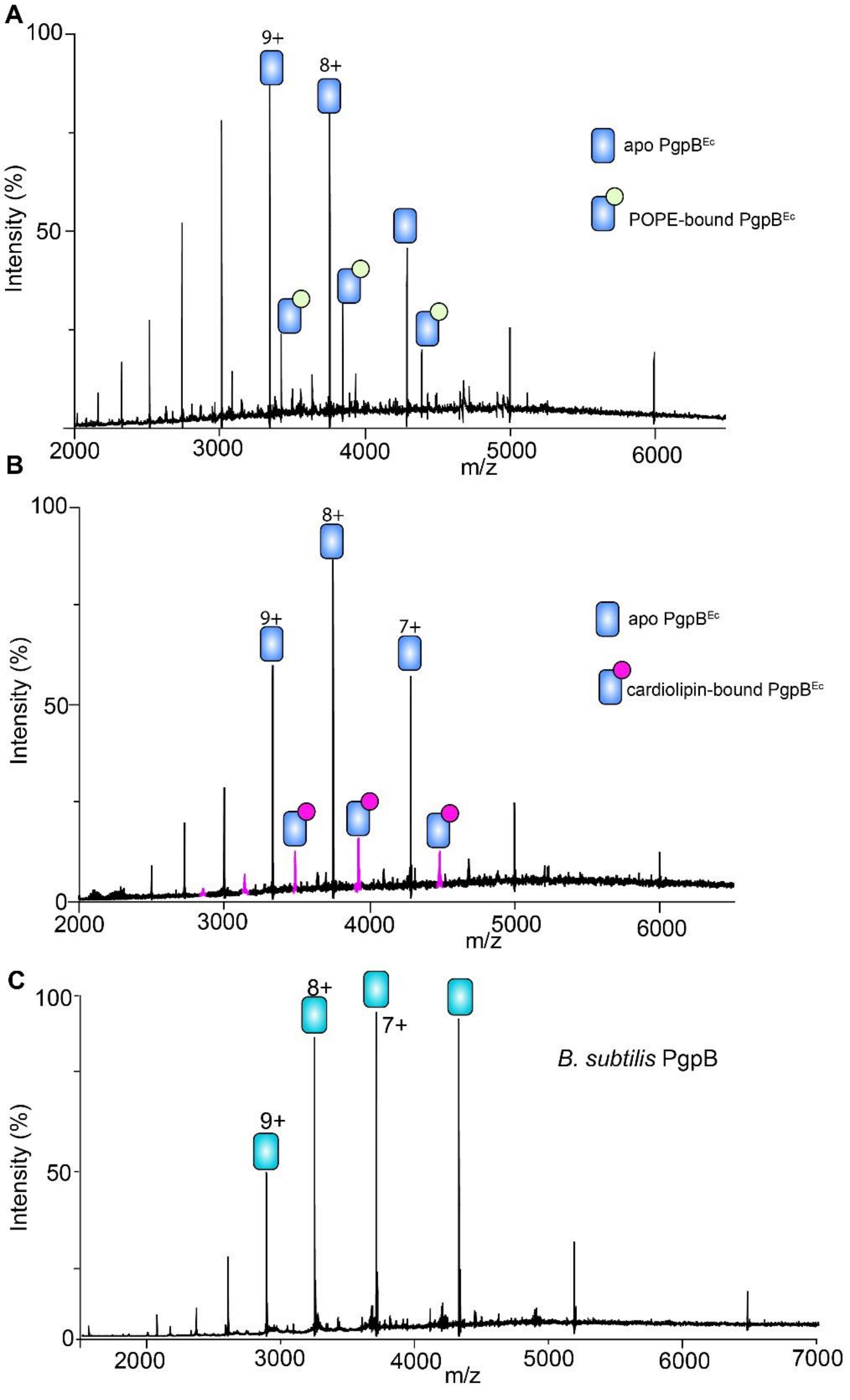

Fig. S3: Native MS of analysis of PgpB released from LDAO micelles. **A**. *E. coli* PgpB (5 µM) titrated with POPE (20 µM). The protein binds POPE but does not form a dimer. **B**. Spectrum of *E. coli* PgpB (5 µM) purified using a lesser amount of 0.5% DDM (see Methods). The protein does not retain endogenous PE, instead, it copurified with cardiolipins. **C.** Mass spectrum of *B. Subtilis* PgpB (5 µM). Like the *E. coli* homologue, this protein copurified with very little phospholipids.

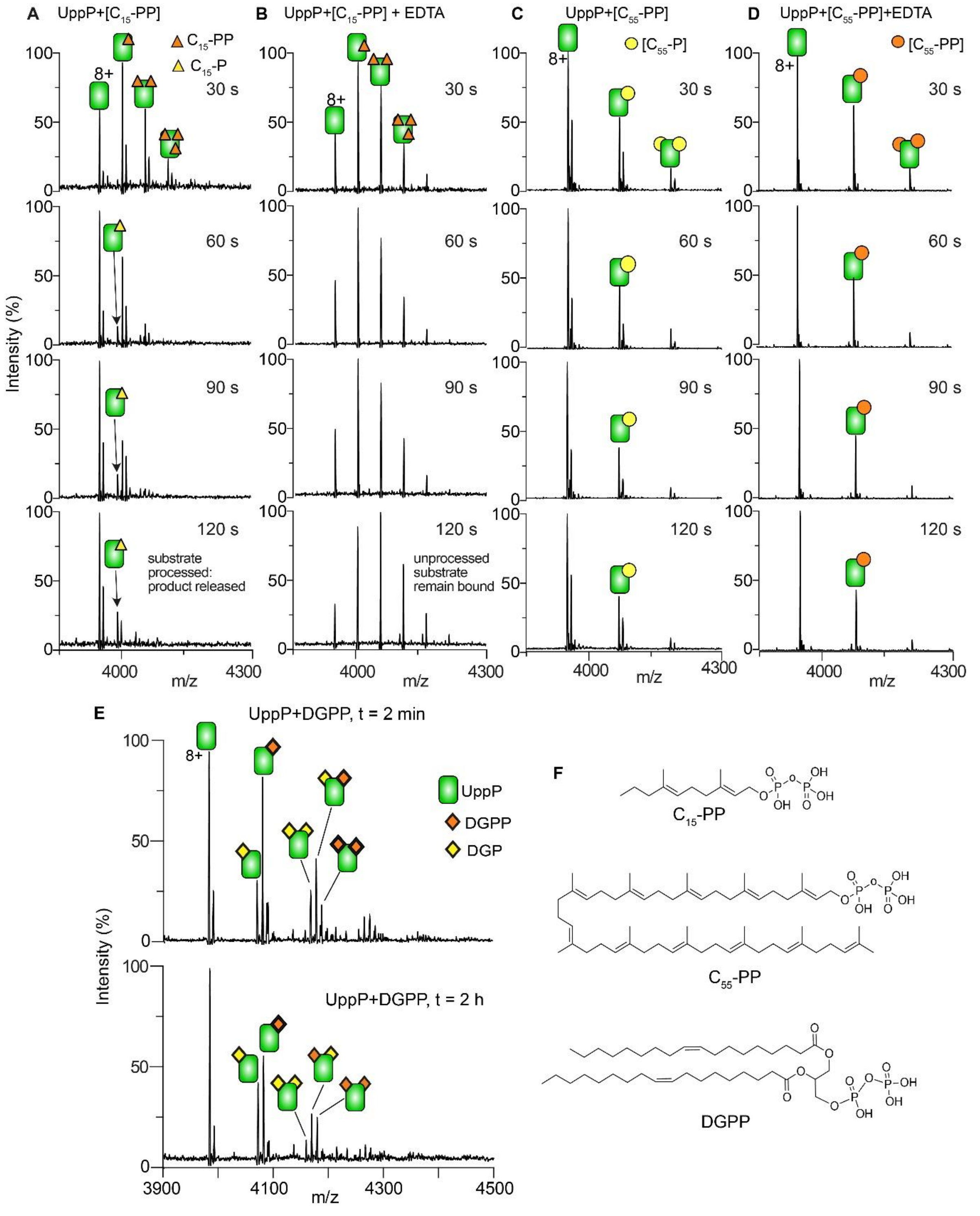

Fig. S4: Real-time monitoring of UppP interactions with C_{15}-PP or C_{55}-PP and inhibition by EDTA. A,B. (8+) charge state of the mass spectra recorded for 3.5 µM UppP equilibrated with 50 µM C_{15}-PP at indicated times in the absence and presence of 200 µM EDTA. The intensity of peaks assigned to enzyme-substrate complexes remained the same in the absence of divalent cations. **C,D**. Equivalent dataset for UppP interaction with 20 µM C_{55}-PP (panel **C**) and inhibition by EDTA (panel **D**). The reaction against C_{55}-PP is completed before the first 30 s. Only bound products are detected. EDTA inhibited UppP function. The absence of product C_{55}-P molecules in the spectra for reactions in the presence of EDTA indicated that UppP is inactive without divalent cations and that the substrates C_{15}-

PP or C_{55}-PP employed in our assay are not contaminated with C_{15}-P or C_{55}-P. For all reactions performed in the absence of EDTA, there are additional peaks in front of each annotated peak that correspond to Ni^{2+} adducts carried over from the purification step of UppP. **E**. Spectra of 3.5 μM UppP equilibrated with 20 μM DGPP acquired at the times indicated. UppP processes DGPP slowly. **F**. Chemical structures of C_{15}-PP, C_{55}-PP, and DGPP.

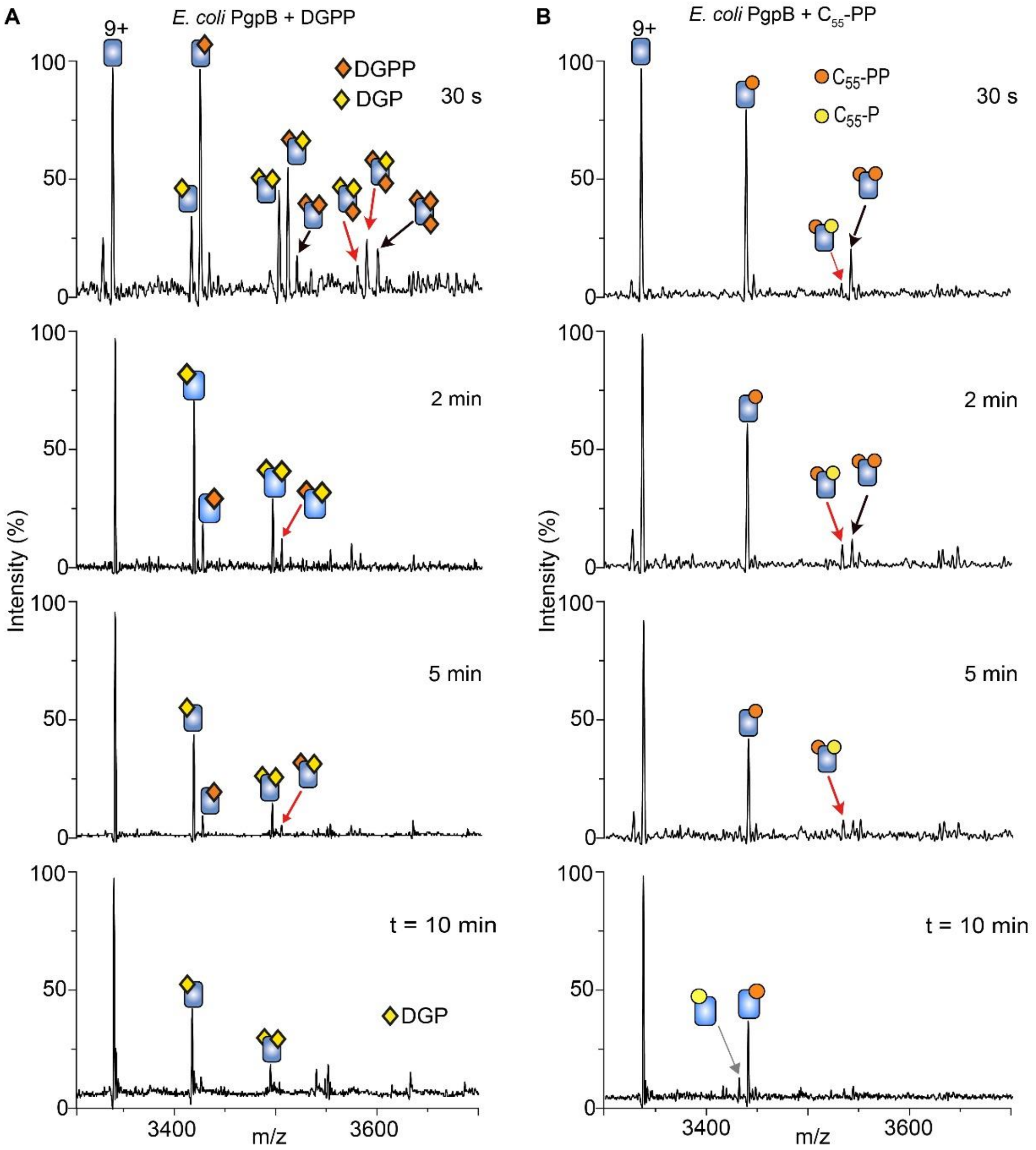

Fig. S5: *E. coli* PgpB processes DGPP faster and more efficiently than C_{55}-PP. A,B. Spectra recorded for 3.5 μM PgpB^{Ec} incubated with 20 μM DGPP (panel A) and 20 μM C_{55}-PP (panel B) as a function of time.

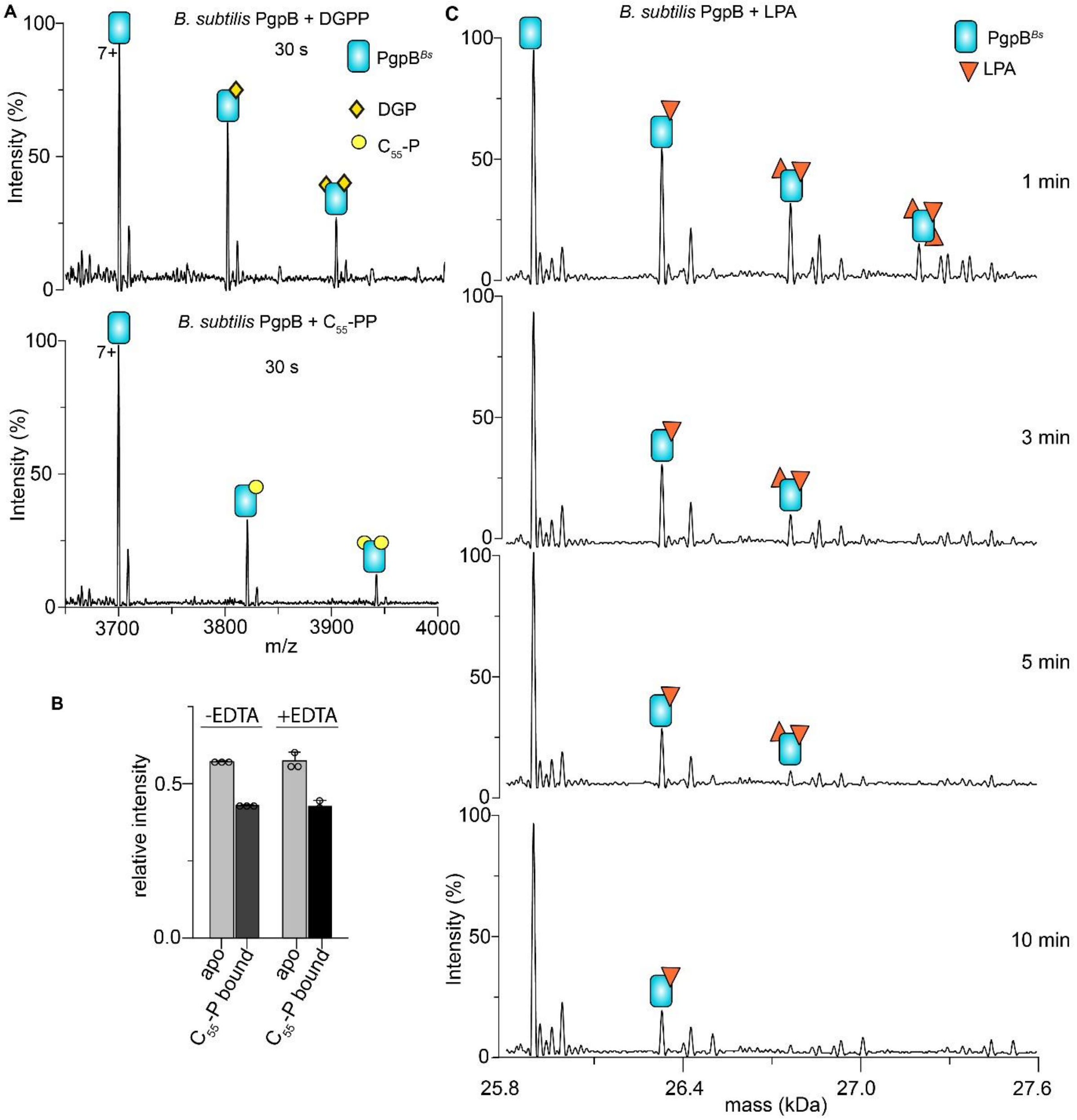

Fig. S6. Activity of *B. Subtilis* PgpB towards C_{55}-PP, DGPP and lysophosphatidic acid (LPA). A. Spectra recorded for 3.5 µM PgpB^{Ec} incubated with 20 µM DGPP (panel A) and 20 µM C_{55}-PP. No residual substrates are detected in the spectra acquired after 30 seconds of mixing protein and the substrate, indicating that *Bacillus Subtilis* PgpB is very efficient in processing both DGPP and C_{55}-PP. **B.** Relative intensity of apo and C_{55}-P bound to PgpB^{Bs} for the reaction performed in the absence and in the presence of 200 µM EDTA. PgpB activity is independent of divalent cations. **C**. Mass spectrum (deconvoluted) for 5 µM PgpB^{Ec} equilibrated with 20 µM LPA. The intensity of the enzyme-substrate complex decreased as a function of time (cf. Fig 2C, main text). Adduct peaks correspond to bound Ni^{2+} (+62 Da) and/or sulphate ions (+98 Da) carried over from protein purification, and LPA stock, respectively.

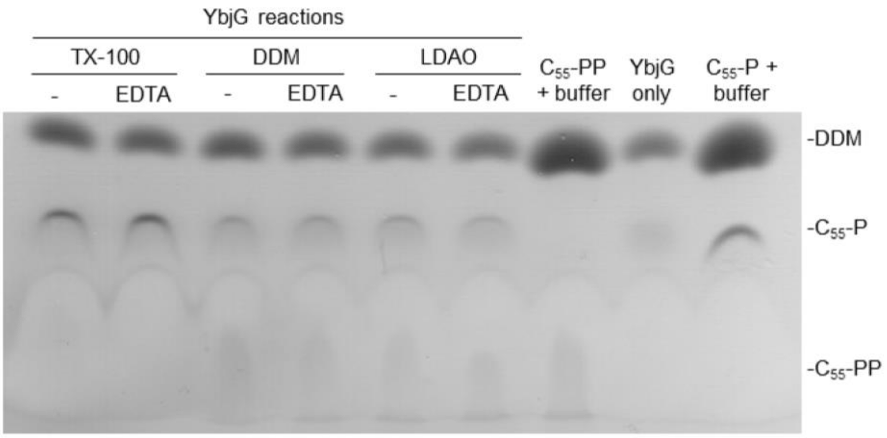

Fig. S7. C_{55}-PP phosphatase activity of YbjG is not sensitive to EDTA. YbjG activity is higher in the presence of Triton X-100 and was not affected by EDTA. TLC analysis of reaction products of C_{55}-PP phosphatase assays with semi-purified YbjG. 0.05 mg/mL of the enzyme was incubated with 31.2 µM C_{55}-PP in the presence or absence of 15 mM EDTA. Reactions were performed in buffer 25 mM Tris pH 7.5, 100 mM NaCl, and 0.2% DDM, with or without additional 0.2% LDAO or Triton X-100. Reactions were incubated for 2 h at 25°C.

A

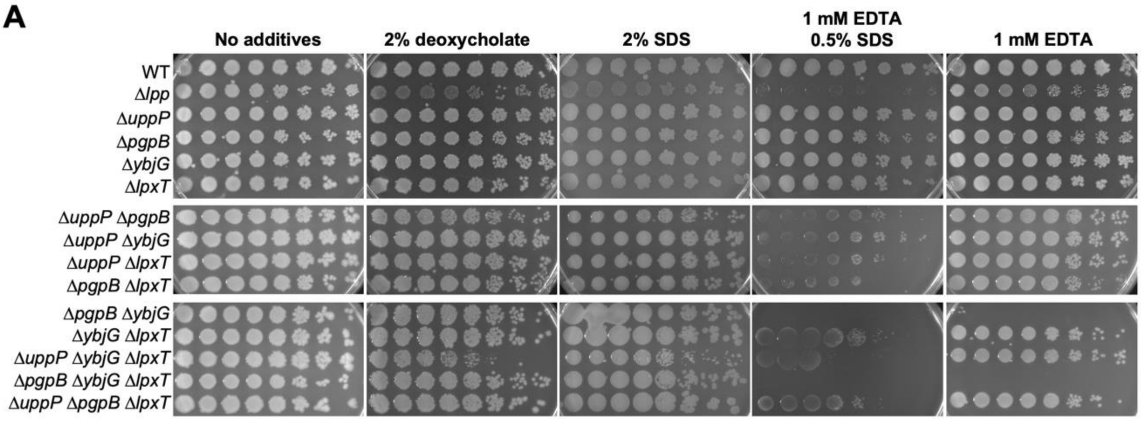

B

Active phosphatases	Strains (BW25113)	Growth in the presence of			
		DOC	SDS	SDS/EDTA	EDTA
All 3	WT	Yes	Yes	Yes	Yes
All 3	Δlpp::kan	Reduced*	Reduced*	No	Yes
PgpB/YbjG	*ΔuppP ΔlpxT*::kan	Yes	Yes	Yes	Yes
UppP/YbjG	*ΔpgpB ΔlpxT*::kan	Yes	Yes	Yes	Yes
UppP/PgpB	*ΔybjG ΔlpxT*::kan	Yes	Yes	Yes	Yes
UppP	*ΔpgpB ΔybjG*::kan	Yes	Yes	No	No
	ΔpgpB ΔybjG ΔlpxT::kan	Yes	Yes	No	No
PgpB	*ΔuppP* ΔybjG::kan	Yes	Yes	Yes	Yes
	ΔuppP ΔybjG ΔlpxT::kan	Reduced	Reduced	Yes	Yes
YgjG	*ΔuppP ΔpgpB*	Yes	Yes	Yes	Yes
	ΔuppP ΔpgpB ΔlpxT::kan	Yes	Yes	Yes	Yes

C

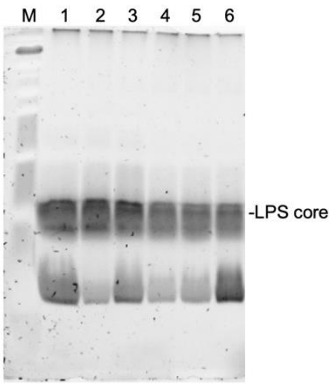

Fig. S8. A. Spot plate assay to test EDTA, SDS and DOC sensitivity of BW25113 *E. coli* strains with different combinations of deletions in *uppP, lpxT, pgpB and ybjG* genes. BW25113 Δ*lpp*::kan, which is known to be sensitive to both EDTA and SDS due to defects in outer membrane attachment to PG, was used as a control. Cells were plated in LB-Agar medium with the indicated additives and incubated for 20 h at 37°C before imaging. B. Summary of the phenotypes observed in panel A. C. LPS analysis of C_{55}-PP deletion strains. LPS analysis for BW25113 strains with deletions in Δ*pgpB* Δ*uppP* (lane 1), Δ*pgpB* Δ*uppP* Δ*lpxT* (lane 2), Δ*pgpB* Δ*ybjG* (lane 3), Δ*pgpB* Δ*ybjG* Δ*lpxT* (lane 4), Δ*upPP* Δ*ybjG* (lane 5), and Δ*uppP* Δ*ybjG* Δ*lpxT* (lane 6). No changes in LPS structure were observed between the different strains.

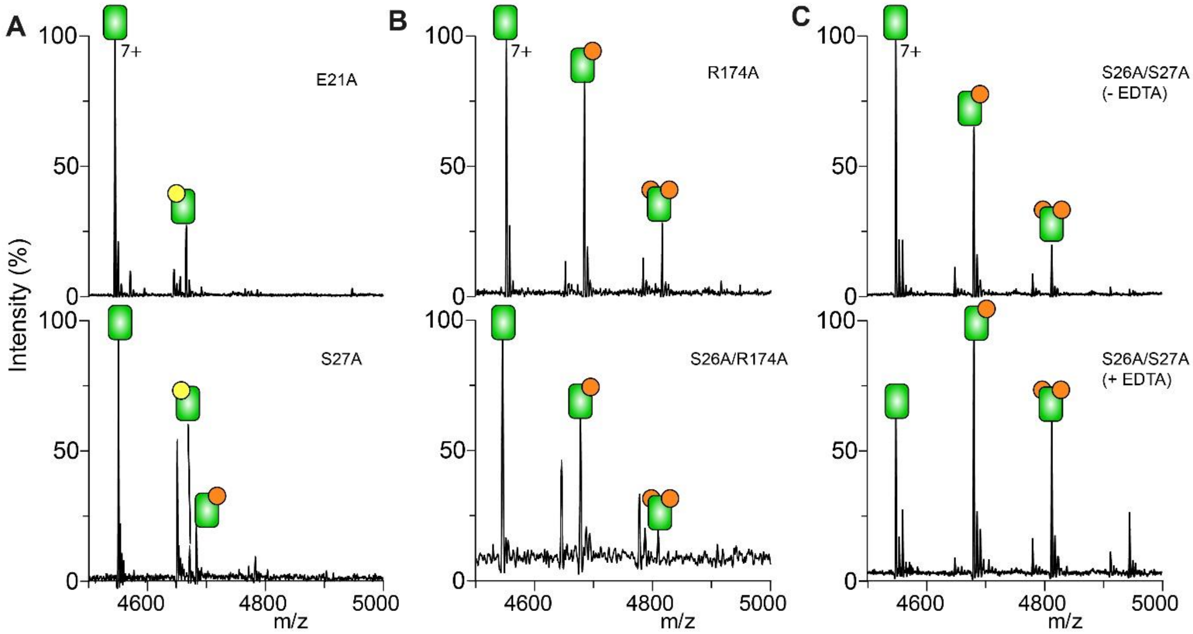

Fig. S9. Native MS (7+ charge state) for different UppP mutants (at 3.5 μM) incubated with 10 μM C_{55}-PP for 30 min in each case. **A**. E21A UppP is active, but only a partial activity was observed for S27A UppP. **B**. R174A UppP and S26A/R174A UppP are inactive. **C**. S26A/S27A UppP is inactive; C_{55}-PP binds to the inactive double mutant more strongly in the presence of EDTA. Expected masses based on amino-acid sequence and the experimentally observed masses for these mutants are included in Table. S4.

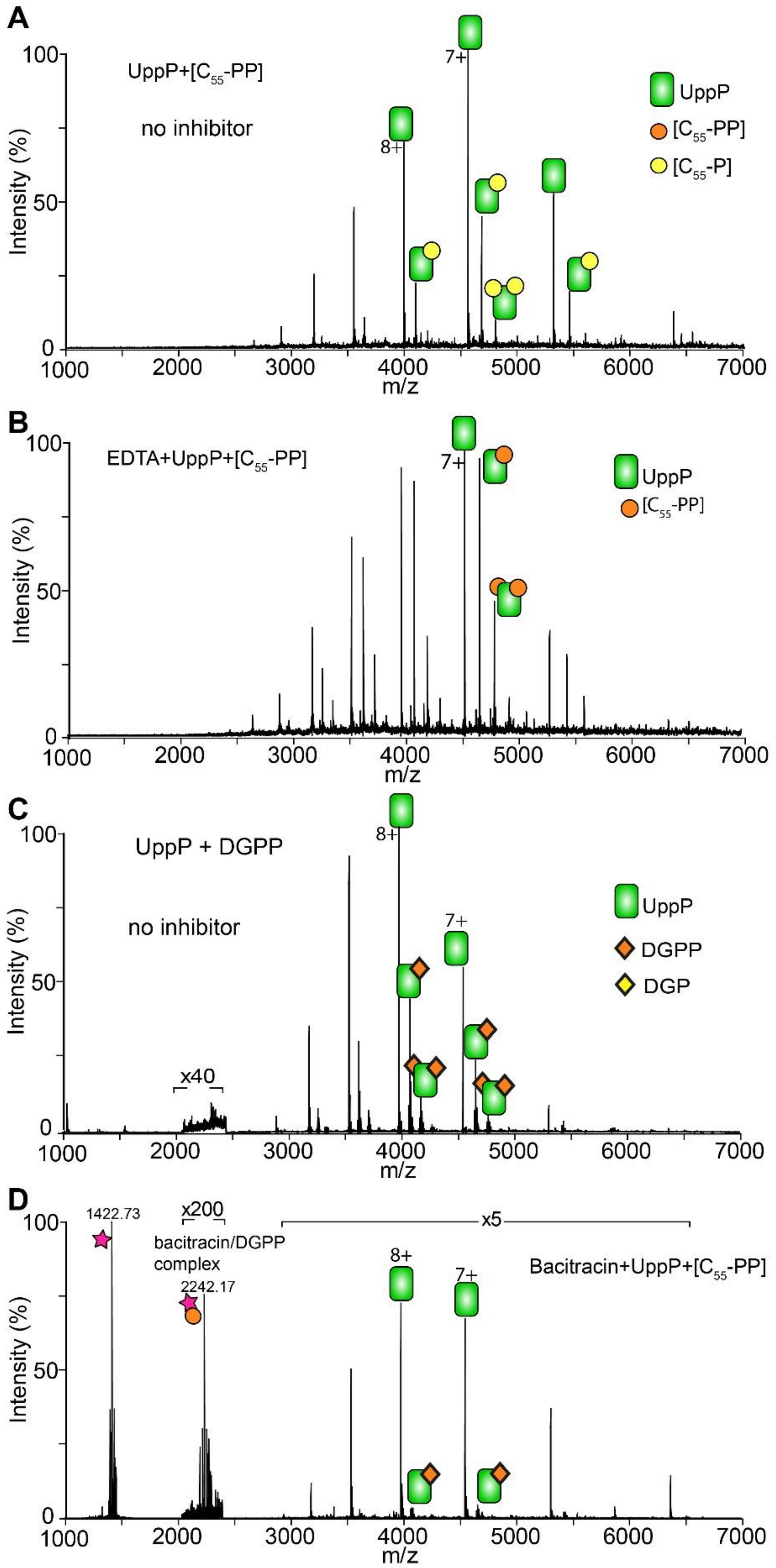

Fig. S10: Effect of EDTA and bacitracin on UppP function. **A,B**. Spectra for a solution containing UppP (3.5μM) incubated with C_{55}-PP (10 μM) with and without EDTA (500 μM) for 30 min. Only Enzyme-product complexes are captured for the uninhibited reaction Removal of divalent cations by EDTA resulted in a lack of UppP activity, and therefore only enzyme-substrate complexes were observed. **C,D.** Equivalent data for UppP activity against DGPP without and with 100 μM bacitracin. The low

intensity of substrates observed in the presence of bacitracin is attributed to bacitracin forming complex with DGPP, effectively inhibiting UppP function.

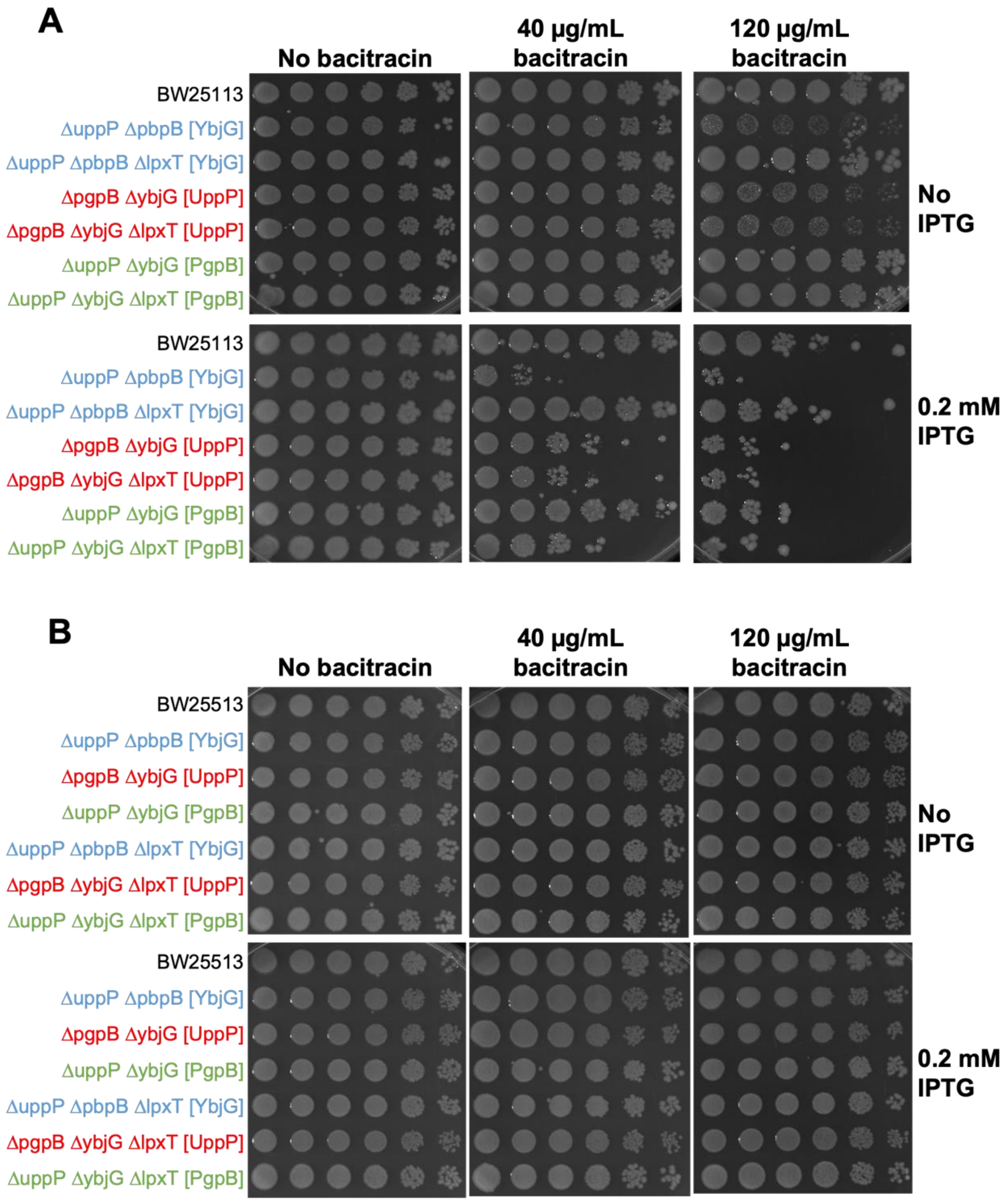

Fig. S11: Cells depending on a single phosphatase have different bacitracin sensitivity. Strains with multiple deletions of C_{55}-PP phosphatases were transformed with either pVMH23 plasmid, encoding the plug-less outer membrane transporter FhuA Δ322-355 (panel A), or the empty plasmid pBB012 (panel B). Growth was monitored on LB-Agar plates containing 0, 40 or 120 µg/mL bacitracin and 0 or 0.2 mM IPTG (to induce FhuA Δ322-355 expression), as indicated. Plates were incubated at 37°C for 24 h. The parental strain, BW25113, was included as a control. In brackets, the name of the remaining C_{55}-PP phosphatase is indicated.

REFERENCES

(1) Baba, T.; Ara, T.; Hasegawa, M.; Takai, Y.; Okumura, Y.; Baba, M.; Datsenko, K. A.; Tomita, M.; Wanner, B. L.; Mori, H. Construction of Escherichia coli K-12 in-frame, single-gene knockout mutants: the Keio collection. *Mol. Syst. Biol.* **2006**, *2*, 2006.0008. DOI: 10.1038/msb4100050.

(2) Silhavy, T. J.; Berman, M. L.; Enquist, L. W. *Experiments with gene fusions*; Cold Spring Harbor Laboratory, 1984.

(3) Datsenko, K. A.; Wanner, B. L. One-step inactivation of chromosomal genes in Escherichia coli K-12 using PCR products. *Proc. Nat.l Acad. Sci. U. S. A.* **2000**, *97* (12), 6640-6645. DOI: 10.1073/pnas.120163297.

(4) Hernández-Rocamora, V. M.; Otten, C. F.; Radkov, A.; Simorre, J. P.; Breukink, E.; VanNieuwenhze, M.; Vollmer, W. Coupling of polymerase and carrier lipid phosphatase prevents product inhibition in peptidoglycan synthesis. *Cell Surf.* **2018**, *2*, 1-13. DOI: 10.1016/j.tcsw.2018.04.002.

(5) Jin, K.; Sam, I. H.; Po, K. H. L.; Lin, D.; Ghazvini Zadeh, E. H.; Chen, S.; Yuan, Y.; Li, X. Total synthesis of teixobactin. *Nat. Commun.* **2016**, *7*, 12394. DOI: 10.1038/ncomms12394.

(6) Marty, M. T.; Baldwin, A. J.; Marklund, E. G.; Hochberg, G. K. A.; Benesch, J. L. P.; Robinson, C. V. Bayesian Deconvolution of Mass and Ion Mobility Spectra: From Binary Interactions to Polydisperse Ensembles. *Anal. Chem.* **2015**, *87* (8), 4370-4376. DOI: 10.1021/acs.analchem.5b00140.

(7) Rick, P. D.; Hubbard, G. L.; Kitaoka, M.; Nagaki, H.; Kinoshita, T.; Dowd, S.; Simplaceanu, V.; Ho, C. Characterization of the lipid-carrier involved in the synthesis of enterobacterial common antigen (ECA) and identification of a novel phosphoglyceride in a mutant of Salmonella typhimurium defective in ECA synthesis. *Glycobiol.* **1998**, *8* (6), 557-567. DOI: 10.1093/glycob/8.6.557.

(8) Schneider, C. A.; Rasband, W. S.; Eliceiri, K. W. NIH Image to ImageJ: 25 years of image analysis. *Nat. Methods* **2012**, *9* (7), 671-675. DOI: 10.1038/nmeth.2089.

(9) Crawford, R. W.; Keestra, A. M.; Winter, S. E.; Xavier, M. N.; Tsolis, R. M.; Tolstikov, V.; Bäumler, A. J. Very long O-antigen chains enhance fitness during Salmonella-induced colitis by increasing bile resistance. *PLoS Pathog.* **2012**, *8* (9), e1002918. DOI: 10.1371/journal.ppat.1002918.

(10) Killmann, H.; Benz, R.; Braun, V. Conversion of the FhuA transport protein into a diffusion channel through the outer membrane of Escherichia coli. *EMBO J.* **1993**, *12* (8), 3007-3016. DOI: 10.1002/j.1460-2075.1993.tb05969.x.

(11) Urner, L. H.; Liko, I.; Yen, H. Y.; Hoi, K. K.; Bolla, J. R.; Gault, J.; Almeida, F. G.; Schweder, M. P.; Shutin, D.; Ehrmann, S.; et al. Modular detergents tailor the purification and structural analysis of membrane proteins including G-protein coupled receptors. *Nat. Commun.* **2020**, *11* (1), 564. DOI: 10.1038/s41467-020-14424-8.
